# Supplementary material for: SAFE, a new therapeutic intervention for families of children with autism: study protocol for a feasibility randomised controlled trial
Source: BMJ Open. 2019 May 27;9(5):e025006. doi: 10.1136/bmjopen-2018-025006 (PMC6549626; doi:10.1136/bmjopen-2018-025006)
Supplement: Author's manuscript [file bmjopen-2018-025006.draft_revisions.pdf]

BMJ Open

BMJ Open is committed to open peer review. As part of this commitment we make the peer review history of every article we publish publicly available.

When an article is published we post the peer reviewers' comments and the authors' responses online. We also post the versions of the paper that were used during peer review. These are the versions that the peer review comments apply to.

The versions of the paper that follow are the versions that were submitted during the peer review process. They are not the versions of record or the final published versions. They should not be cited or distributed as the published version of this manuscript.

BMJ Open is an open access journal and the full, final, typeset and author-corrected version of record of the manuscript is available on our site with no access controls, subscription charges or pay-per-view fees (http://bmjopen.bmj.com).

If you have any questions on BMJ Open's open peer review process please email info.bmjopen@bmj.com

BMJ Open

SAFE, a new therapeutic intervention for families of children with autism: study protocol for a feasibility randomised controlled trial

Journal:	*BMJ Open*
Manuscript ID	bmjopen-2018-025006
Article Type:	Protocol
Date Submitted by the Author:	25-Jun-2018

Complete List of Authors:	McKenzie, Rebecca; University of Plymouth, Institute of Education<br>Dallos, Rudi; Department of Clinical Psychology, University of Plymouth<br>Hancocks, Helen<br>Ewings, Paul; Research Design Service, Research Office<br>Barton, Andy; South West Research Design Service<br>Vassallo, Tara<br>Myhill, Craig

Keywords:	autism, family therapy, intervention, feasibility

SCHOLARONE™ Manuscripts

SAFE, a new therapeutic intervention for families of children with autism: study protocol for a feasibility randomised controlled trial

Rebecca McKenzie[1], Rudi Dallos[1], Helen Hancocks[2], Paul Ewings[3], Andy Barton[3], Tara Vassallo[1], Craig Myhill[1]

Author affiliations

1. Plymouth University. Plymouth UK
2. Peninsular Clinical Trials Unit. Plymouth UK
3. Regional Design Service. South West. UK

Correspondence to: Dr Rebecca McKenzie rebecca.mckenzie@plymouth.ac.uk

Key words: Family Therapy, autism, intervention, feasibility, mental health

Word count: 4267 excluding title page, abstract and references

For peer review only

ABSTRACT

Introduction: Incidence of autistic traits, mental health problems, stress and poor coping skills is high among family members of children with autism. These problems are coupled with maladaptive behaviour among the children with autism. Current treatment for these families is disjointed and costly. The need for s the whole family support is supported by NICE recommendations, developments regarding children's service provision, research in this field and requests by families of children with autism. Despite evidence that family therapies can provide benefits to these families, its efficacy has not been subject to a randomised controlled trial. SAFE is a new family therapy intervention designed specifically for families of children with autism. We aim to establish the feasibility of running a fully powered randomised controlled trial to evaluate SAFE.

Methods and analysis: Families of children with autism aged 3-6 years will be invited to participate. Consenting participants will be randomised 2:1 to either SAFE + support as usual or support as usual alone. The primary outcome measure will be the Systemic CORE 15. Participants will also complete secondary outcome measures, which index changes in child behaviour, child-parent attachment, anxiety, and depression. Generic health economic outcome measures (EQ-5D-5L and CHU-9D) will also provide data on the feasibility of cost effectiveness analysis. Questionnaires will be completed at baseline and 32 weeks post allocation. Data on ability to identify, recruit, randomise, retain and collect data from families, acceptability of outcome measures, adherence of therapists and families to the intervention, appropriateness of resource use questionnaires and effectiveness of training will be collected for feasibility analysis. Qualitative data will also explore acceptability of SAFE and reasons for declining and withdrawing from the study.

Ethics and dissemination: The current trial protocol received ethical approval from the South West-Exeter Research Ethics Committee (Ref: 17/SW/0192).

Trial registration number: ISCTRN83964946 IRAS 213527

For peer review only - http://bmjopen.bmj.com/site/about/guidelines.xhtml

STRENGTHS AND LIMITATIONS OF THIS STUDY

- The study addresses a gap in the available research data, and will produce important feasibility information to inform a fully powered randomised controlled trial.
- The study explores the feasibility of using measures of family function and a range of mental health measures.
- Quantitative feasibility data are complemented by qualitative focus groups and interviews.
- The study explores the feasibility of economic analysis measures in a population, which includes adults and their children with developmental disorders.
- The participants are recruited from two NHS Trusts in adjacent counties in the South West of England, leading to potential bias. A future randomised controlled trial will extend to centres across the UK including Scotland and Wales.

INTRODUCTION

More than 1% of the UK population has a diagnosis of autism and numbers are rising. Families of children with autism present complex needs. Children with autism have impairments in social interaction, communication, and behavioural flexibility [1]. Autism is widely accepted to have a genetic component and the Broad Autism Phenotype is disproportionally represented among family members [2]. Mental health problems are experienced by more than 70% of individuals with autism and more than 50% of their parents [3,4]. Individuals with autism suffer from high levels of anxiety and depression compared to those with other developmental conditions [5,6,7]. Families of children with autism have higher rates of depression, anxiety, and social phobia, than families with typically developing children, or children with other developmental disorders [8]. Parents of children with autism are more likely to be hospitalised for mental disorders than parents of typically developing children [9]; and mothers of children with autism are reported to have higher unmet needs, more difficulties coping, and lower satisfaction with service interactions than mothers of children with other disabilities [10].

As families of children with autism often exhibit psychological morbidities alongside autism, costs of services to treat these problems are high [11,12]. Furthermore, untreated or unresponsive mental health problems impose societal costs making it hard for parents to interact effectively with services [13], worsening outcomes for children, and exacerbating the substantial economic burden of autism.

For peer review only - http://bmjopen.bmj.com/site/about/guidelines.xhtml

Explanations for high levels of affective disorders in these families include: stress associated with the condition of autism, genetic factors, and intergenerational family dynamics. Parenting children with autism involves stresses associated with maladaptive behaviour, lack of empathy, and atypical attachment behaviour displayed by children [14]. Studies exploring the medical histories of family members indicate, however, that onset of affective disorders predate the birth of the child [8,15,16] suggesting that mental health difficulties cannot be wholly accounted for by stress involved in parenting. Depression and anxiety among family members have been tentatively linked to genetic factors independent of the Broad Autism Phenotype [17]. Few studies explore the intergenerational presence of affective disorder associated with autism and more work is needed in this area [8,15,16]. In particular, there are few studies, which question whether affective disorders combined with autistic traits among parents, pose an environmental risk for the development of autistic symptoms in children. Limited work demonstrates, however, that a child's risk of developing autism is doubled if both parents have a personality or psychiatric disorder [9]; and parents of children with autism report that lack of psychological wellbeing exacerbates maladaptive behaviour in their children [18]. A related body of research exists in the attachment literature, which suggests that emotional problems displayed by the parents of children with autism may be trans-generationally transmitted through insecure attachment patterns in families.

Our initial work [19] indicates that these parents have frequently experienced high levels of trauma and subsequent mental health problems. Unresolved trauma is known to impede parenting abilities and is associated with the development of severe forms of pathology in children. In addition, these families often encounter difficulties communicating needs to external agencies [20], which may trigger existing tendencies for negative affect. This is borne out by studies, reporting that families of children with autism are often characterised by a palpable air of tension [21]. Families of children with autism represent a high-risk group, yet treatment for these families is disjointed, costly and inadequate [22,23].

A more joined-up approach is required which focuses on autism related need, coping with maladaptive behaviour and mental health difficulties by encouraging fundamental reflective functioning and improving family dynamics. The SAFE study should be placed in the context of NICE guidelines and recommendations [24,25] as well as developments regarding children's service provision proposed by the Munroe Report [26,27], and the 'Future in Mind' children and young people's mental health report [28]. The SAFE study also reflects recommendations by other researchers working in the field [29,30]. Families of children with autism themselves highlight the importance of professionals working therapeutically with children and the wider family, in contrast to parents of children with conditions such as Down Syndrome who tend to stress the support needs of their child within educational and community settings [10].

SAFE is a systemic family therapy approach designed by experts to address autism related needs including mental health difficulties and problematic behaviour. Systemic Family Therapy is a well-recognized, evidence-based psychotherapeutic approach [31], which is

For peer review only - http://bmjopen.bmj.com/site/about/guidelines.xhtml

recommended treatment for conditions such as Conduct Disorder, ADHD and Anorexia Nervosa [32]. Despite evidence that family therapies can provide benefits to children with autism and their parents [33,34] its efficacy for treating this condition has not been subject to a randomised controlled trial. A comprehensive search of clinical trial registries revealed no on-going trials assessing Systemic Family Therapy as a treatment for autism and associated mental health problems. This is surprising given guidelines and recommendations for care; the successful use of family therapies for a range of conditions and reports documenting key areas of concern for the UK autism community [35,36], which overwhelmingly show that families of children with autism want interventions which make real improvements to their daily life and sense of wellbeing. Consequently, the overarching aim of this study is to establish the feasibility of a definitive randomised controlled trial to evaluate Systemic Autism-related Family Enabling (SAFE) therapy for families of children with autism.

METHODS AND ANALYSIS

Participants and recruitment

Our target population are families of children with autism, who do not have an intellectual disability, between the ages of 3 and 16 years. Participants will be identified and recruited from two study research sites: Plymouth Hospitals NHS Trust (PHNT) Child Development Centre, and Royal Cornwall Hospitals NHS Trust Autism Spectrum Disorder Assessment Team (ASDAT). The pathways used to identify and recruit families will vary according to local practice, and the needs of the individual families being approached. Some families will receive a diagnosis during the SAFE recruitment period, and others will have been diagnosed up to 12 months before the SAFE study recruitment period starts.

Families with a diagnosis during the SAFE recruitment period will be approached by the diagnosing paediatrician, who will perform an initial eligibility check, invite the families to find out more and, if interested, refer the family to a member of the local SAFE study team. Families with a diagnosis before the SAFE study recruitment period will be identified as potentially eligible from clinic records by a suitably qualified member of the clinical team at each centre. All potential participant families will receive a participant information leaflet including an invitation to take part. All interested families will be able to speak to a member of the study team to discuss the study and have any questions answered.

The participant information leaflet will contain information about the study in plain English. Parents will be asked to explain the information to younger children in a way that is appropriate for their child and suggestions for how to do this will be contained in the leaflet. A home visit will be arranged by a member of the study team for those families who express interest in participating. During the visit, a research assistant will provide the families with more detailed participant information and seek consent.

For peer review only - http://bmjopen.bmj.com/site/about/guidelines.xhtml

Inclusion criteria

- Family includes child with ASD, aged 3-16 years
- Diagnosis of autism spectrum disorder, severity level 1 or 2
- Diagnosed within 12 months of consenting to the study
- If other diagnoses are present, ASD must be primary diagnosis
- Family are willing to comply with study requirements

Exclusion criteria

- Children with ASD severity level 3
- Children with ASD and intellectual impairment
- Serious concomitant illness in child or family, or other circumstances such that they are unable to comply with study requirements
- Families who may be a risk to safety of research staff
- Insufficient English language or capacity for parent/child to consent/assent to the study.

Study design

This is a randomised, controlled, multi-centred feasibility study including children with autism and their families. Four cohorts of 9 families totalling 36 families will be recruited and each cohort will be randomised in a 2:1 ratio to receive support as usually employed (SUE) plus a programme of Systemic Autism-related Family Enabling (SAFE) therapy, or SUE alone, for a period of 16 weeks. Outcome assessors will be blinded to allocation. All participants will complete outcome measures at baseline and again at 32 weeks post-allocation via a face-to-face visit, hence each family will participate in the SAFE study for approximately eight months. The end date for the trial will be the date on which the last family completes the 32-week follow-up visit. An embedded qualitative study will collect information about the feasibility and acceptability of the intervention and the study itself.

Outcome measures

Feasibility outcome measures:

- Ability to identify, recruit and randomise eligible families.
- Acceptability of proposed outcome measures and follow-up schedule to participants, and whether targets for loss to follow-up are achievable.
- Adherence of therapists and families to the intervention.
- Ability to gather quantitative data on outcomes
- Appropriateness of resource use questionnaires and preference-based instruments for this population
- Effectiveness and scalability of training arrangements

For peer review only - http://bmjopen.bmj.com/site/about/guidelines.xhtml

Clinical outcome measures:

- Scores on the proposed primary outcome measure, the Systemic CORE 15 (SCORE) [37]. This is a 15 item paper-based survey, which is a reliable, valid index of family functioning, and takes approximately 20 minutes to complete. SCORE is the primary measure of family functioning employed in CYP (Children and Young People's) Improving Access to Psychological Therapies national programme, and is the gold standard for assessing the impact of family therapy on quality of life in the UK [38]. Every able family member will be asked to complete the SCORE, and the same family members should complete the SCORE at baseline and 32 weeks.

- Scores on the proposed secondary outcome measures, which index changes in child behaviour, child-parent attachment, anxiety, and depression.
  - Patient Health Questionnaire – Somatic Anxiety Depressive Symptoms (PHQ-SADS). This comprises the PHQ-9 measuring depression and the GAD-7 measuring anxiety [39].
  - Coding of Attachment-Related Parenting for use with children with Autism - CARP-A [40]. The CARP-A is an observational measure of child's attachment behaviour towards their carer.
  - The Child Behaviour Checklist (CBCL) [41]. This is a 30-item paper-based survey, which detects emotional and behavioural problems.
  - The Reflective Functioning Questionnaire (RFQ) [42] measures ability to understand own and others' mental states.
  - Caregiving Helplessness Questionnaire [43] (CGHQ). This is a 26-item questionnaire designed to assess aspects of disorganised caregiving.

- Scores on generic health economic outcome measures will also provide data on the feasibility of cost effectiveness analysis:
  - EuroQol 5 dimensions (EQ-5D-5L). This is a standardised generic instrument for measuring health outcome.
  - Child Health Utility 9 Dimensions (CHU-9D). The CHU-9D is a paediatric generic preference based measure of health related quality of life.
  - Resource Use Questionnaire. A paper-based questionnaire completed by parent about his/her child's use of health care and social resources.

- Qualitative outcomes:
  - Acceptability of SAFE and the trial process for participants and therapists
  - Reasons for declining and withdrawing from the study

For peer review only - http://bmjopen.bmj.com/site/about/guidelines.xhtml

The qualitative component will employ focus groups and interviews to investigate four key aspects of the study experience: families' experiences of the study (including intervention), therapists' experiences of the intervention, reasons for eligible families declining, and reasons for families withdrawing from the study. After the 32-week assessments have been completed, participating families that have consented to participate in the qualitative focus groups will be given details of the time and location for the family feedback day. The family feedback day will involve several separate focus group sessions organised to take place over the period of a morning or an afternoon at a local venue for each centre including focus groups aimed specifically at parents and at children.

The Intervention

SAFE is a manualised intensive programme of systemic family therapy designed to treat maladaptive autistic symptoms and mental health related difficulties encountered by families of children with autism. SAFE provides a toolkit of therapeutic activities based on Attachment Theory, established systemic practice and the known visual processing preferences of people with autism. Each therapy session will include two therapists with a minimum of intermediate family therapy level of qualification and four days training in SAFE principles. Between weeks 1 and 16, families allocated to the SAFE intervention will attend five 3-hour SAFE therapy sessions. Sessions 1 and 5 are multi-family sessions and will take place in a community setting. Sessions 2, 3 and 4 are for individual families and will take place in a community venue or the family home. The therapists will facilitate sessions which will be video recorded, as is usual practice for therapy sessions. The videotapes will be used by the therapists in supervision sessions and preparation for subsequent sessions.

Following completion of the therapy programme, families will attend a group follow-up session at 24 weeks post-allocation. Trained support workers from local voluntary groups will attend this follow-up session and will be invited to give the families information about continued support for families of children with autism through existing networks.

Each session will include the following assessments for families to complete:

1. Client Satisfaction Questionnaire (CSQ-8)
2. The Helpful Aspects of Therapy Questionnaire (HAT)
3. A Between Session Activity (BSA) homework activity Families will be given a pro-forma with key elements of the intervention as prompts for families to track strengths and difficulties in response to SAFE ideas.

At the end of each session the therapists will also complete a training checklist and questionnaire to monitor protocol adherence.

Support as usually employed

For peer review only - http://bmjopen.bmj.com/site/about/guidelines.xhtml

Families will typically be offered a post-diagnosis follow-up appointment with the diagnosing paediatrician. Parents of children whose symptoms are not severe may be directed to local authority parenting classes. Classes such as 'Timeout for Autism' (Cornwall and the Isles of Scilly) and 'Understanding Autism' (Plymouth) focus on the features of Autism Spectrum Disorder, instructional parenting techniques and issues associated with education. Psycho-education may also be offered, with families being directed to relevant resources for e.g. The National Autism Society, Gateway ASD, and the NHS Child and Adolescent Mental Health Services (CAMHS). For families where a member is experiencing depression or anxiety, treatment varies and is not linked to autism-related care. Initial referral is often through the GP. Patients may receive Cognitive Behavioural Therapy as part of the Improved Access for Psychological Therapies service. They may also receive medication and in extreme cases a period of in-patient hospital treatment.

Proposed sample size

In this feasibility trial no formal statistical testing of between group differences is planned. Sample size has been selected heuristically with the goal of i) demonstrating that participants can be recruited at a rate sufficient to run a full scale evaluation of SAFE at a later date; ii) demonstrating that it is possible to train therapists and deliver SAFE to patients within the study treatment settings, and iii) demonstrate that the data collection procedures are effective, and that the data collection is acceptable to the 36 families, and not overly burdensome.

Data analysis

Completed paper case report forms will be checked and signed by research staff before being sent to the Clinical Trials Unit (CTU). Original case report form pages will be posted to the CTU at agreed time points for double-data entry on to a password-protected database, with copies retained at the study site. Forms will be tracked using a web-based trial management system. Data will be analysed and presented as is appropriate for a feasibility study, in particular concentrating on descriptive analyses and undertaking no formal comparisons between groups. Reporting will follow the principles of the CONSORT Statement using the checklist and flowchart as recommended in the CONSORT extension for Randomized Pilot and Feasibility [44]. The flowchart will provide detail about the number of families approached, number eligible, number consenting, number randomised, number receiving allocated intervention and number assessed for outcome data at each time point. As appropriate, details will be given for individual members of the family, for example, how many family members there are and how many completed each questionnaire. Wherever possible, detailed reasons will be given for exclusions, loss to follow-up, non-completion of outcome measures etc.

Numbers will also be provided by centre and group, to inform the logistics of recruiting nine families prior to randomisation and following them up after randomisation. For those

For peer review only - http://bmjopen.bmj.com/site/about/guidelines.xhtml

randomised to the SAFE intervention, adherence will be reported according to the number of group sessions attended and participation of individual family members at each of the therapy sessions. Completeness of data will be reported for each outcome measure at each relevant time point. Again this will be reported for individual family members as appropriate.

For each outcome measure, the relevant scores will be calculated and presented descriptively by trial arm. Where available, published guidelines will be used to process, score and summarise the measures including, for example, the use of imputation in the event of missing items on a questionnaire. Summary measures will be calculated as appropriate, for example, means and standard deviations, medians and ranges, numbers and percentages in categories. These measures will be presented both for baseline and for the final follow-up. The only analysis contrasting the two groups will be an interval estimate in the form of a 95% confidence interval for the primary outcome, so that the plausibility for the effect size used in the sample size calculation for the full trial can be assessed. For this purpose the baseline values will be used in an Analysis of Covariance, with acknowledgment that no effects are included for group or therapist.

Focus group interviews will be audio recorded and transcribed verbatim. Consequent qualitative data will be managed using proprietary computer assisted qualitative data analysis software, for example, Nvivo 10, and analysed thematically. Rigour of analysis will include 'respondent validation', whereby participants are provided with a summary of their transcript and analysis so that they can assess whether the interpretations being made about the data, accurately represent them. In addition, a second qualitative researcher will conduct an independent analysis of a subset of half of the focus group transcripts. Researchers will then meet to discuss and agree the findings, which will then be presented to the Family Consultation Group for discussion.

ETHICS AND DISSEMINATION

Ethics and Safety

Families of children with autism are a vulnerable group. The risks associated with participating in this study are however, considered minimal, with no adverse events anticipated in any participant. For those in the intervention group, there is a slight chance that the SAFE family therapy sessions could lead to an initial increase in family disagreements as family members learn how to change the way they solve problems and talk with one another. However, the purpose of the intervention is ultimately to equip families with skills to handle these difficulties by learning how to change the way they solve problems and improve their communication, and the SAFE family therapists will be available to provide support and will be trained to handle any emerging problems. Should any issues arise the SAFE family therapists will have access to two consultant clinical psychologists to provide further support and advice.

For peer review only - http://bmjopen.bmj.com/site/about/guidelines.xhtml

During the trial the children with autism will remain in the care of the Child Development Centre or the Autism Spectrum Disorder Assessment Team and will have access to usual care should any unforeseen circumstances arise. Other members of the family will also continue to be able to seek care and advice from the GP or any other specialist services they are concurrently involved with.

Informing potential participants of possible benefits and known risks

The participant information sheets and leaflets will provide potential participants with information about the possible benefits and risks of taking part in the trial. For example, the participants will be informed that a potential risk of receiving the SAFE therapy is that the sessions may evoke difficult emotions and feelings this could lead to family disagreement as they move towards change. The families will also be informed that benefits of the trial include the possibility of improved coping skills when faced with challenges and contribution to finding out if SAFE can progress to a national trial. Participants will be given the opportunity to discuss risks and benefits with a member of the research team prior to consenting to participate.

Obtaining Informed consent from participants

All participants will receive a leaflet and information sheet prior to consent. There are two versions of the information sheet, one for adults and one for children. In the leaflet parents are encouraged to explain the trial to their younger children and some guidance for doing this is provided. The information sheet states that the participants have the right to withdraw at any point during the trial and that data collected from them will be confidential. All participants will have a home-visit prior to consent from a member of the research team and will be able to ask questions or go through the information verbally.

Data protection/confidentiality

Participants will be given an identification number. The research team will ensure that participants' pseudo-anonymity is maintained on all documents. Data will be collected and stored in accordance with the current legal and regulatory documentation. Electronic study records will be stored in a SQL server database, stored on a restricted access, secure server maintained by Plymouth University. Data will be entered into the database via a bespoke web-based data entry system encrypted using SSL. Access to electronic data will be permission based, and at the discretion of the clinical trials unit data management team.

Anonymised paper-based study data will be stored in locked filing cabinets within a locked office. Copies of study data retained at the lead study site will be securely stored for the duration of the study prior to archiving. Video data will be transported via encrypted memory

For peer review only - http://bmjopen.bmj.com/site/about/guidelines.xhtml

sticks and will be transferred to a password-protected computer. The clinical trials unit data team will have access to study data, including identifiable data. Other members of the study team and the trials unit will have restricted access to pseudo-anonymised study data. Access will be granted to the Sponsor and host institution on request, to permit study-related monitoring, audits and inspections.

Research governance and the conduct of the trial

The trial will be conducted to protect the human rights and dignity of the participant as reflected in the Helsinki Declaration. An important factor in protecting the participants is ongoing consultation with the SAFE Family Consultation Group. A representative of this group is a member of the research team and is involved in decision-making processes. The research team including the family consultation group are proactive in minimising discomfort and risk for participants, respecting their wishes over science and society, respecting the right to withdraw and the need for families to have access to all relevant information.

Dissemination plans

If the feasibility study demonstrates successful recruitment and an ability to deliver the intervention an important part the dissemination plan is to raise awareness of the need for a larger multi-centre trial. Targeted summaries of the findings and presentations will be disseminated to policy makers. The findings will also be broadly disseminated, but in a manner appropriate to a feasibility study. National conference presentations and published papers will be prepared to inform clinicians, academics and therapists about our feasibility results and generate interest in the future trial. Existing connections including the Association for Family Therapy, the National Autistic Society and the Institute of Family Therapy will be utilised to reach relevant audiences. The qualitative findings will also be published with detailed accounts of the families' reactions to SAFE and their views on its usefulness. A summary of study results in plain English will be available on the Peninsula Clinical Trials website.

Clinical trials authorisation and ethical approval

Clinical trials authorisation is not required. The study has appropriate Research Ethics Committee (REC) approval from the South West-Exeter Research Ethics Committee (Ref: 17/SW/0192) and approval from the Health Research Authority (HRA). The trial will be conducted in accordance with the protocol, the principles of the Declaration of Helsinki and ICH GCP. Any amendments of the protocol will be submitted to the Sponsor, HRA and REC for approval.

For peer review only - http://bmjopen.bmj.com/site/about/guidelines.xhtml

Trial sponsorship

The trial is sponsored by Plymouth Hospitals NHS Trust.

Monitoring adverse events

The research team have mechanisms in place to report serious adverse events (SAE) related to mental health. If the CI considers that the SAE is not, or is unlikely to be, associated with the trial, the clinical trials unit will obtain a second assessment of causality from an independent assessor. Any SAE which in the opinion of either adjudicator is possibly related to the trial will be reported to the Research Ethics Committee within 15 days of the local research team having become aware of the event. All SAEs will be followed until either stabilised if chronic conditions or resolved.

Trial Steering Committee

The Trial Steering Committee will include an independent chair and at two other independent members, along with the lead investigator and the other study collaborators including a parent representative. They will meet once a year.

REFERENCES

1. American Psychiatric Association. Diagnostic and Statistical Manual of Mental Disorders, 5th edn. American Psychiatric Association, Washington, DC 2013.

2. Sasson NJ, Lam KSL, Parlier M, et al. Autism and the broad autism phenotype: familial patterns and intergenerational transmission. *J Neurodev Disord* 2013; 5:11-18.

3. Dykens EM, Fisher MH, Taylor JL, et al. Reducing Distress in Mothers of Children with Autism and Other Disabilities: A Randomized Trial. *Pediatrics* 2014; 2013-3164.

4. Hallett V, Ronald A, Colvert, E, et al. Exploring anxiety symptoms in a large-scale twin study of children with autism spectrum disorders, their co-twins and controls. *Journal of Child Psychology and Psychiatry* 2013;54:1176-1185.

5. Brereton AV, Tonge BJ, Einfeld SL. Psychopathology in children and adolescents with autism compared to young people with intellectual disability. *J Autism Dev Disord* 2006;36: 863-870.

6. Kim JA, Szatmari P, Bryson SE, et al. The prevalence of anxiety and mood problems among children with autism and Asperger syndrome. *Autism* 2000; 4:117-132.

For peer review only - http://bmjopen.bmj.com/site/about/guidelines.xhtml

7. Howlin P. Interventions for people with autism: recent advances. *Adv Psychiatr Treat* 1997;3: 94-102.

8. Mazefsky CA, Folstein SE, Lainhart JE. Overrepresentation of Mood and Anxiety Disorders in Adults with Autism and Their First Degree Relatives: What Does it Mean? *Autism Res* 2008;1:193-197.

9. Daniels JL, Forssen U, Hultman M, et al. Parental Psychiatric Disorders Associated With Autism Spectrum Disorders in the Offspring. *Pediatrics* 2008;121:1357-1162.

10. Siklos S, Kerns KA. Assessing Need for Social Support in Parents of Children with Autism and Down Syndrome. *J Autism Dev Disord* 2008;36:921-933.

11. McCrone P, Dhanasiri S, Patel A, et al. The cost of mental health care in England to 2026. Executive Summary, London: The King's Fund. 2008

12. Buescher AVS, Cidav Z, Knapp M, et al. Costs of Autism Spectrum Disorders in the United Kingdom and the United States. *JAMA Pediatr* 2014;e1-e8.

13. Osborne LA, McHugh L, Saunders J, et al. Parenting Stress Reduces the Effectiveness of Early Teaching Interventions for Autistic Spectrum Disorders. *J Autism Dev Disord* 2008;38:1092-1103.

14. Pisula E. Parenting Stress in Mothers and Fathers of Children with Autism Spectrum Disorders, A Comprehensive Book on Autism Spectrum Disorders, Dr. Mohammad-Reza Mohammadi (Ed.) 2011. Available from: http://www.intechopen.com/books/a-comprehensive- bookon- autism-spectrum-disorders/parenting-stress-in-mothers-and-fathers-of-children-with-autismspectrum-disorders

15. Bolton PF, Pickles A, Murphy M, et al. Autism, affective and other psychiatric disorders: patterns of familial aggregation. *Psychological Medicine* 1998;28: 385-395.

16. Demir T, Dogangun B, Karacetin G, et al. Psychiatric Disorders and Symptoms in Mothers of Children with Autistic Disorder. *Neurol Psychiatry Brain Res* 2008;15:1-5.

17. Piven J, Palmer P. Psychiatric disorder and the broad autism phenotype: evidence from a family study of multiple-incidence autism families. *Am J Psychiatry* 1999;156:557-563.

18. Nikmat AW, Ahmad M, Oon NL, et al. Stress and psychological wellbeing among parents of children with autism spectrum disorder. *ASEAN Journal of Psychiatry* 2008;9:65-72.

19. McKenzie R, Dallos R, Autism and Family Dynamics. Manuscript in preparation.

For peer review only - http://bmjopen.bmj.com/site/about/guidelines.xhtml

20. Konstantareas MK. Autistic, learning disabled and delayed children's impact on their parents. *Can J Behav Sci* 1991;23:358-375.

21. Murphy M, Bolton PF, Pickles A, et al. Personality traits of the relatives of autistic probands. *Psychol Med* 2000;30:1411-1424.

22. Blackledge JT, Hayes SC. Using Acceptance and Commitment Training in the Support of Parents of Children Diagnosed with Autism. *Child & Family Behavior Therapy,* 2006:28:1-18.

23. Davis G. Lead Paediatric Psychologist, Plymouth Hospitals Trust. Personal communication, September 10, 2014.

24. NICE guidelines [CG170] https://www.nice.org.uk/guidance/cg170/chapter/patient-centred-care

25. NICE guidelines [CG128] https://www.nice.org.uk/guidance/cg128

26. Munroe E. The Munroe Review of Child protection: Final report, Dept. of Education, HM Stationary Office, UK 2011.

27. Goodman S, Trowler I. Social work re-claimed: Innovative frameworks for child and social work Practice. London: Jessica Kingsley 2012.

28. NHS England. Future in Mind: promoting, protecting and improving our children and young people's mental health and wellbeing. Dept of Health Publication Gateway Ref. No 02939, 2015.

29. IAPT | CYP IAPT Children and Young People's Project. (n.d.). Retrieved January 5, 2015, from http://www.iapt.nhs.uk/cyp-iapt/

30. Bromley J, Hare DJ, Davison K. et al. Mothers supporting children with autistic spectrum disorders: Social support, mental health status and satisfaction with services. *Autism* 2004;8:409-423.

31. Stratton P. Report On The Evidence Base Of Systemic Family Therapy. *Association for Family Therapy* 2005.

32. Dallos R, Draper R. (4th Edn) An Introduction to Family Therapy. Maidenhead: Open Univ. Press/McGraw Hill 2015.

33. Monteiro M. Family Therapy and the Autism Spectrum: Autism Conversations in Narrative Practice. London: Routledge 2016.

For peer review only - http://bmjopen.bmj.com/site/about/guidelines.xhtml

34. Estrada AU, Pinsof WF. The effectiveness of family therapies for selected behavioural disorders of childhood. *J Marital Fam Ther* 1995;21: 403-424.

35. Pellicano E, Dinsmore A, Charman T. What should autism research focus upon? Community views and priorities from the United Kingdom. *Autism, Special Issue Article* 2014:1-15.

36. Wallace S, Parr J, Hardy, A. One in a hundred: Putting families at the heart of autism research. London: Autistica 2013.

37. Stratton P, Bland J, Janes E, et al. Developing an indicator of family function and a practicable outcome measure for systemic family and couple therapy: the SCORE. *Journal of Family Therapy* 2010;32:232-258.

38. SHIFT Self-Harm Intervention, Family Therapy: a randomised controlled trial of family therapy vs. treatment as usual for young people seen after second or subsequent episodes of self harm. Protocol Grant Number: 470772 HTA Reference Number: 07/33/01, 2015.

39. Spitzer R L, Kroenke K, Williams JBW. The Patient Health Questionnaire Primary Care Study Group. Validation and utility of a self-report version of PRIME-MD: the PHQ Primary Care Study. *JAMA,* 282, 1999:1737-1744.

40. Blazey L. Coding of Attachment-Related Parenting for use with children with Autism (CARP-A). Manchester University 2007.

41. Moss E, Bureau J, Cyr C, et al. Correlates of attachment at age 3 Construct validity of the Preschool Attachment Classification System. *Dev. Psychol* 2004;40:323-334.

42. Fonagy P, Luyten P, Moulton-Perkins A, et al. Development and validation of a self-report measure of mentalizing: The Reflective Functioning Questionnaire. PLOS ONE. 2016;11(7):e0158678.

43. Solomon J, George C. (Eds.) Disorganised Attachment and Caregiving*.* Guilford: New York 2011.

44. CONSORT extension for Randomized Pilot and Feasibility Trials (http://www.consort-statement.org/randomised-pilot-and-feasibilitytrials).

AUTHORS' CONTRIBUTIONS: RM and HH were responsible for the overall development of the protocol. RM, HH, RD, CM, PE, AB and TV were involved in the conception and production of the study and the development of the initial protocol. PE and AB provided methodological expertise and advice on quantitative analysis PE provided statistical

For peer review only - http://bmjopen.bmj.com/site/about/guidelines.xhtml

expertise. RD was the lead researcher on design of the intervention and the qualitative component. TV and CM advised on design and ethics, particularly from the participant perspective. All authors made substantial contributions to drafting, revision and approval of the document.

FUNDING STATEMENT: This work was supported by the National Institute for Health Research (NIHR) grant number: PB-PG-0815-20058

COMPETING INTERESTS STATEMENT: Contributors are co-applicants or employed research staff on the SAFE project, which receives funds from both NIHR and Autistica. Professor Rudi Dallos holds joint Intellectual Property rights for the SAFE intervention with The University of Plymouth. No other issues were raised with regard to competing interests.

PROVENANCE AND PEER REVIEW: Not commissioned; externally peer reviewed.

DATA SHARING STATEMENT: Further details of the study protocol can be requested from the corresponding author.

For peer review only

STANDARD PROTOCOL ITEMS: RECOMMENDATIONS FOR INTERVENTIONAL TRIALS

SPIRIT 2013 Checklist: Recommended items to address in a clinical trial protocol and related documents*

Section/item	Item No	Description	Addressed on page number
**Administrative information**			
Title	1	Descriptive title identifying the study design, population, interventions, and, if applicable, trial acronym	Front page
Trial registration	2a	Trial identifier and registry name. If not yet registered, name of intended registry	Front page
	2b	All items from the World Health Organization Trial Registration Data Set	Front page, 6, 9
Protocol version	3	Date and version identifier	Front page
Funding	4	Sources and types of financial, material, and other support	34
Roles and responsibilities	5a	Names, affiliations, and roles of protocol contributors	6
	5b	Name and contact information for the trial sponsor	6
	5c	Role of study sponsor and funders, if any, in study design; collection, management, analysis, and interpretation of data; writing of the report; and the decision to submit the report for publication, including whether they will have ultimate authority over any of these activities	5
	5d	Composition, roles, and responsibilities of the coordinating centre, steering committee, endpoint adjudication committee, data management team, and other individuals or groups overseeing the trial, if applicable (see Item 21a for data monitoring committee)	30 - 32

For peer review only

For peer review only - http://bmjopen.bmj.com/site/about/guidelines.xhtml

**Introduction**			
Background and rationale	6a	Description of research question and justification for undertaking the trial, including summary of relevant studies (published and unpublished) examining benefits and harms for each intervention	10 -11
	6b	Explanation for choice of comparators	29 - 30
Objectives	7	Specific objectives or hypotheses	11 - 12
Trial design	8	Description of trial design including type of trial (eg, parallel group, crossover, factorial, single group), allocation ratio, and framework (eg, superiority, equivalence, noninferiority, exploratory)	12
**Methods: Participants, interventions, and outcomes**			
Study setting	9	Description of study settings (eg, community clinic, academic hospital) and list of countries where data will be collected. Reference to where list of study sites can be obtained	16
Eligibility criteria	10	Inclusion and exclusion criteria for participants. If applicable, eligibility criteria for study centres and individuals who will perform the interventions (eg, surgeons, psychotherapists)	16
Interventions	11a	Interventions for each group with sufficient detail to allow replication, including how and when they will be administered	21 - 22
	11b	Criteria for discontinuing or modifying allocated interventions for a given trial participant (eg, drug dose change in response to harms, participant request, or improving/worsening disease)	21
	11c	Strategies to improve adherence to intervention protocols, and any procedures for monitoring adherence (eg, drug tablet return, laboratory tests)	21, 30
	11d	Relevant concomitant care and interventions that are permitted or prohibited during the trial	22
Outcomes	12	Primary, secondary, and other outcomes, including the specific measurement variable (eg, systolic blood pressure), analysis metric (eg, change from baseline, final value, time to event), method of aggregation (eg, median, proportion), and time point for each outcome. Explanation of the clinical relevance of chosen efficacy and harm outcomes is strongly recommended	14 - 15
Participant timeline	13	Time schedule of enrolment, interventions (including any run-ins and washouts), assessments, and visits for participants. A schematic diagram is highly recommended (see Figure)	19

For peer review only - http://bmjopen.bmj.com/site/about/guidelines.xhtml

Sample size	14	Estimated number of participants needed to achieve study objectives and how it was determined, including clinical and statistical assumptions supporting any sample size calculations	29 - 30
Recruitment	15	Strategies for achieving adequate participant enrolment to reach target sample size	20
**Methods: Assignment of interventions (for controlled trials)**			
Allocation:			
Sequence generation	16a	Method of generating the allocation sequence (eg, computer-generated random numbers), and list of any factors for stratification. To reduce predictability of a random sequence, details of any planned restriction (eg, blocking) should be provided in a separate document that is unavailable to those who enrol participants or assign interventions	29 - 30
Allocation concealment mechanism	16b	Mechanism of implementing the allocation sequence (eg, central telephone; sequentially numbered, opaque, sealed envelopes), describing any steps to conceal the sequence until interventions are assigned	19 - 20
Implementation	16c	Who will generate the allocation sequence, who will enrol participants, and who will assign participants to interventions	20
Blinding (masking)	17a	Who will be blinded after assignment to interventions (eg, trial participants, care providers, outcome assessors, data analysts), and how	20 - 21
	17b	If blinded, circumstances under which unblinding is permissible, and procedure for revealing a participant's allocated intervention during the trial	20 - 21
**Methods: Data collection, management, and analysis**			
Data collection methods	18a	Plans for assessment and collection of outcome, baseline, and other trial data, including any related processes to promote data quality (eg, duplicate measurements, training of assessors) and a description of study instruments (eg, questionnaires, laboratory tests) along with their reliability and validity, if known. Reference to where data collection forms can be found, if not in the protocol	27 - 29
	18b	Plans to promote participant retention and complete follow-up, including list of any outcome data to be collected for participants who discontinue or deviate from intervention protocols	24 - 25

For peer review only - http://bmjopen.bmj.com/site/about/guidelines.xhtml

Data management	19	Plans for data entry, coding, security, and storage, including any related processes to promote data quality (eg, double data entry; range checks for data values). Reference to where details of data management procedures can be found, if not in the protocol	28
Statistical methods	20a	Statistical methods for analysing primary and secondary outcomes. Reference to where other details of the statistical analysis plan can be found, if not in the protocol	29 - 30
	20b	Methods for any additional analyses (eg, subgroup and adjusted analyses)	25 - 26
	20c	Definition of analysis population relating to protocol non-adherence (eg, as randomised analysis), and any statistical methods to handle missing data (eg, multiple imputation)	29 - 30
**Methods: Monitoring**			
Data monitoring	21a	Composition of data monitoring committee (DMC); summary of its role and reporting structure; statement of whether it is independent from the sponsor and competing interests; and reference to where further details about its charter can be found, if not in the protocol. Alternatively, an explanation of why a DMC is not needed	31 - TSC performing DMC role.
	21b	Description of any interim analyses and stopping guidelines, including who will have access to these interim results and make the final decision to terminate the trial	33
Harms	22	Plans for collecting, assessing, reporting, and managing solicited and spontaneously reported adverse events and other unintended effects of trial interventions or trial conduct	26 - 27
Auditing	23	Frequency and procedures for auditing trial conduct, if any, and whether the process will be independent from investigators and the sponsor	30 - 31
**Ethics and dissemination**			
Research ethics approval	24	Plans for seeking research ethics committee/institutional review board (REC/IRB) approval	32 - 33
Protocol amendments	25	Plans for communicating important protocol modifications (eg, changes to eligibility criteria, outcomes, analyses) to relevant parties (eg, investigators, REC/IRBs, trial participants, trial registries, journals, regulators)	32

For peer review only - http://bmjopen.bmj.com/site/about/guidelines.xhtml

Consent or assent	26a	Who will obtain informed consent or assent from potential trial participants or authorised surrogates, and how (see Item 32)	18
	26b	Additional consent provisions for collection and use of participant data and biological specimens in ancillary studies, if applicable	23
Confidentiality	27	How personal information about potential and enrolled participants will be collected, shared, and maintained in order to protect confidentiality before, during, and after the trial	28 - 29
Declaration of interests	28	Financial and other competing interests for principal investigators for the overall trial and each study site	______________
Access to data	29	Statement of who will have access to the final trial dataset, and disclosure of contractual agreements that limit such access for investigators	29
Ancillary and post-trial care	30	Provisions, if any, for ancillary and post-trial care, and for compensation to those who suffer harm from trial participation	33
Dissemination policy	31a	Plans for investigators and sponsor to communicate trial results to participants, healthcare professionals, the public, and other relevant groups (eg, via publication, reporting in results databases, or other data sharing arrangements), including any publication restrictions	33 - 34
	31b	Authorship eligibility guidelines and any intended use of professional writers	______________
	31c	Plans, if any, for granting public access to the full protocol, participant-level dataset, and statistical code	______________
**Appendices**			
Informed consent materials	32	Model consent form and other related documentation given to participants and authorised surrogates	______________
Biological specimens	33	Plans for collection, laboratory evaluation, and storage of biological specimens for genetic or molecular analysis in the current trial and for future use in ancillary studies, if applicable	N/A

*It is strongly recommended that this checklist be read in conjunction with the SPIRIT 2013 Explanation & Elaboration for important clarification on the items. Amendments to the protocol should be tracked and dated. The SPIRIT checklist is copyrighted by the SPIRIT Group under the Creative Commons "Attribution-NonCommercial-NoDerivs 3.0 Unported" license.

For peer review only - http://bmjopen.bmj.com/site/about/guidelines.xhtml

BMJ Open

SAFE, a new therapeutic intervention for families of children with autism: study protocol for a feasibility randomised controlled trial

Journal:	*BMJ Open*
Manuscript ID	bmjopen-2018-025006.R1
Article Type:	Protocol
Date Submitted by the Author:	08-Oct-2018
Complete List of Authors:	McKenzie, Rebecca; University of Plymouth, Institute of Education<br>Dallos, Rudi; Department of Clinical Psychology, University of Plymouth<br>Hancocks, Helen<br>Vickery, Patricia; Plymouth University, Peninsula Clinical Trials Unit<br>Ewings, Paul; Research Design Service, Research Office<br>Barton, Andy; South West Research Design Service<br>Vassallo, Tara<br>Myhill, Craig
**Primary Subject Heading**:	Mental health
Secondary Subject Heading:	Public health, Pathology, Research methods
Keywords:	autism, family therapy, intervention, feasibility

SCHOLARONE™ Manuscripts

SAFE, a new therapeutic intervention for families of children with autism: study protocol for a feasibility randomised controlled trial

Rebecca McKenzie[1], Rudi Dallos[1], Helen Hancocks[2], Jane Vickery[2], Paul Ewings[3], Andy Barton[3], Tara Vassallo[1], Craig Myhill[1]

Author affiliations

1. Plymouth University. Plymouth UK
2. Peninsula Clinical Trials Unit, Plymouth University. Plymouth UK
3. NIHR Research Design Service. South West. UK

Correspondence to: Dr Rebecca McKenzie rebecca.mckenzie@plymouth.ac.uk

Key words: Family Therapy, autism, intervention, feasibility, mental health

Word count: 5903 excluding title page, abstract and references

For peer review only

ABSTRACT

Introduction: Incidence of autistic traits, mental health problems, stress and poor coping is high among family members of children with autism. These problems are coupled with challenging behaviour among children with autism. Current treatment for these families is disjointed and costly. The need for whole family support is supported by NICE recommendations, developments regarding children's service provision, research and requests by families of children with autism. Despite evidence that family therapies can provide benefits to these families, its efficacy has not been subject to a randomised controlled trial. SAFE is a new family therapy intervention designed specifically for families of children with autism. We aim to establish the feasibility of running a fully powered randomised controlled trial to evaluate SAFE.

Methods and analysis: Families of children with autism aged 3-16 years will be invited to participate. Consenting participants will be randomised 2:1 to either SAFE + support as usual or support as usual alone. The proposed primary outcome measure for the main trial will be the Systemic CORE 15. Participants will also complete proposed secondary outcome measures, indexing changes in child behaviour, child-parent attachment, anxiety, and depression. Generic health economic outcome measures (EQ-5D-5L and CHU-9D) will also provide data on the feasibility of cost effectiveness analysis. Questionnaires will be completed at baseline and 32 weeks post allocation. Data on ability to identify, recruit, randomise, retain and collect data from families, acceptability of outcome measures, adherence of therapists and families to the intervention, appropriateness of resource use questionnaires and effectiveness of training will be collected for feasibility analysis. Qualitative data will also explore acceptability of SAFE and reasons for declining and withdrawing from the study.

Ethics and dissemination: The current trial protocol received ethical approval from the South West-Exeter Research Ethics Committee (Ref: 17/SW/0192).

Trial registration number: ISCTRN83964946 IRAS 213527

For peer review only - http://bmjopen.bmj.com/site/about/guidelines.xhtml

STRENGTHS AND LIMITATIONS OF THIS STUDY

- The study addresses a gap in the available research data, and will produce important feasibility information to inform a fully powered randomised controlled trial.
- The study explores the feasibility of using measures of family function and a range of mental health measures.
- Quantitative feasibility data are complemented by qualitative focus groups and interviews.
- The study explores the feasibility of economic analysis measures in a population, which includes adults and their children with developmental disorders.
- The participants are recruited from two NHS Trusts in adjacent counties in the South West of England, leading to potential bias. A future randomised controlled trial will extend to centres across the UK including Scotland and Wales.

INTRODUCTION

More than 1% of the UK population has a diagnosis of autism [1]. Families of children with autism present complex needs. Children with autism have impairments in social interaction, communication, and behavioural flexibility [2]. Autism is widely accepted to have a genetic component and the Broad Autism Phenotype is disproportionally represented among family members [3]. Mental health problems are experienced by more than 70% of individuals with autism and more than 50% of their parents [4,5]. Individuals with autism suffer from high levels of anxiety and depression compared to those with other developmental conditions [6,7,8]. Families of children with autism have higher rates of depression, anxiety, and social phobia, than families with typically developing children, or children with other developmental disorders [9]. Parents of children with autism are more likely to be hospitalised for mental disorders than parents of typically developing children [10]; and mothers of children with autism are reported to have higher unmet needs, more difficulties coping, and lower satisfaction with service interactions than mothers of children with other disabilities [11]. Aside from these reported difficulties, families of children with autism can have positive family experiences, sense of wellbeing [12] and positive perceptions of their children [13]. Despite challenges, autism can be seen as enhancing family experience and some parents recognise that parenting a child with autism has added joy to their lives [14], made them more appreciative [15], more patient and compassionate [16].

As families of children with autism often exhibit psychological morbidities alongside autism, costs of services to treat these problems are high [17,18]. Furthermore, untreated or unresponsive mental health problems impose societal costs making it hard for parents to interact effectively with services [19], potentially worsening outcomes for children and exacerbating the substantial economic burden of autism [18].

For peer review only - http://bmjopen.bmj.com/site/about/guidelines.xhtml

Explanations for high levels of affective disorders in these families include: stress associated with the condition of autism, genetic factors, and intergenerational family dynamics. Parenting children with autism involves stresses associated with challenging behaviour, lack of Theory of Mind, and atypical attachment behaviour displayed by children [20]. Studies exploring the medical histories of family members indicate, however, that onset of affective disorders predate the birth of the child [9,21,22] suggesting that mental health difficulties cannot be wholly accounted for by stress involved in parenting. Depression and anxiety among family members have been tentatively linked to genetic factors independent of the Broad Autism Phenotype [23]. Few studies explore the intergenerational presence of affective disorder associated with autism and more work is needed in this area [9,21,22]. In particular, there are few studies which question whether affective disorders combined with autistic traits among parents pose an environmental risk for the development of autistic symptoms in children. Limited work demonstrates, however, that a child's risk of developing autism is doubled if both parents have a personality or psychiatric disorder [11]; and parents of children with autism report that lack of psychological wellbeing exacerbates maladaptive behaviour in their children [24].

Previous studies demonstrate that experience of trauma and abuse among women is associated with elevated risk of autism developing in their subsequent offspring [25,26]. Hence mothers of children with autism are more likely than the general population to be coping with previous traumatic events. Unresolved trauma is known to impede parenting abilities and is associated with the development of severe forms of pathology in children. In addition, these families often encounter difficulties communicating needs to external agencies [27], which may trigger existing tendencies for negative affect. Families of children with autism can experience positive family life, cope well with difficulties and enjoy good relationships with their children but they represent a high-risk group, for whom treatment is disjointed, costly and inadequate [28,29].

A more joined-up approach is required which focuses on autism related need, coping with challenging behaviour and mental health difficulties by encouraging fundamental reflective functioning and improving family dynamics. The SAFE study should be placed in the context of NICE guidelines and recommendations [30,31] as well as developments regarding children's service provision proposed by the Munroe Report [32,33], and the 'Future in Mind' children and young people's mental health report [34]. The SAFE study also reflects recommendations by other researchers working in the field [35,36]. Families of children with autism themselves highlight the importance of professionals working therapeutically with children and the wider family, in contrast to parents of children with conditions such as Down Syndrome who tend to stress the support needs of their child within educational and community settings [10].

SAFE is a systemic family therapy approach designed by experts to address autism related needs including mental health difficulties and problematic behaviour. Systemic Family Therapy is a well-recognized, evidence-based psychotherapeutic approach [37], which is recommended treatment for conditions such as Conduct Disorder, ADHD and Anorexia

For peer review only - http://bmjopen.bmj.com/site/about/guidelines.xhtml

Nervosa [38]. Despite evidence that family therapies can provide benefits to children with autism and their parents [39,40] its efficacy for treating this condition has not been subject to a randomised controlled trial. A comprehensive search of clinical trial registries revealed no on-going trials assessing Systemic Family Therapy as a treatment for autism and associated mental health problems. This is surprising given guidelines and recommendations for care; the successful use of family therapies for a range of conditions and reports documenting key areas of concern for the UK autism community [41,42], which overwhelmingly show that families of children with autism want interventions which make real improvements to their daily life and sense of wellbeing. Consequently, the overarching aim of this study is to establish the feasibility of a definitive randomised controlled trial to evaluate Systemic Autism-related Family Enabling (SAFE) therapy for families of children with autism.

METHODS AND ANALYSIS

Participants and recruitment

Our target population are families of children with autism, who do not have an intellectual impairment, between the ages of 3 and 16 years. SAFE is designed to have a visual play-based approach, but children gain most from the intervention if they can understand and communicate their responses to SAFE activities. Pilot data suggest that SAFE will be most effective and accessible for children who do not have severe symptoms or an intellectual impairment. Those children who were non-verbal and/or had severe communication difficulties found it difficult to engage with some activities. For this feasibility study, therefore, our target population is families of children with autism severity level 1 or 2 with no intellectual impairment. Future plans for SAFE include the development of a sister intervention which has extended non-verbal elements based on Intensive Interaction and is designed specifically to support families of children with autism and an intellectual impairment. This feasibility study focuses on families of children of school-age which fits with the priorities of one of our secondary sources of funding. Background research exploring diagnostic data for our proposed centres for the previous two years revealed no children without intellectual impairment diagnosed before the age of 3 years. Consequently, we focused on the 3-16 age group.

Participants will be identified and recruited from two study research sites: University Hospitals Plymouth NHS Trust (PHNT) Child Development Centre, and Cornwall Partnership NHS Foundation Trust Autism Spectrum Disorder Assessment Team (ASDAT). The pathways used to identify and recruit families will vary according to local practice, and the needs of the individual families being approached. Some families will receive a diagnosis during the SAFE recruitment period, and others will have been diagnosed up to 12 months before the SAFE study recruitment period starts.

Families with a diagnosis during the SAFE recruitment period will be approached by the

For peer review only - http://bmjopen.bmj.com/site/about/guidelines.xhtml

diagnosing paediatrician, who will perform an initial eligibility check, invite the families to find out more and, if interested, refer the family to a member of the local SAFE study team. Families with a diagnosis before the SAFE study recruitment period will be identified as potentially eligible from clinic records by a suitably qualified member of the clinical team at each centre. Clinical staff in our centres and the surrounding areas are responsible for diagnosis of the children within our participating families. If the child is recruited from a diagnostic centre the clinical staff also assess eligibility.

All potential participant families will receive a participant information leaflet including an invitation to take part. All interested families will be able to speak to a member of the study team to discuss the study and have any questions answered. The participant information leaflet will contain information about the study in plain English. Parents will be asked to explain the information to younger children in a way that is appropriate for their child and suggestions for how to do this will be contained in the leaflet. A home visit will be arranged by a member of the study team for those families who express interest in participating. During the visit, a research assistant will provide the families with more detailed participant information and seek consent.

Community pathway

Participants that have received either a new diagnosis, or a diagnosis within the last 12 months will also be approached through community groups, using a recruitment poster, invitation letter, reply slip, participant information leaflet, and freepost envelope. These participants will be contacted by a member of the research team by telephone at which time they will discuss the study and answer questions. The families will also be asked to consent to providing the original NHS diagnosis letter, which will be used by the research staff to determine eligibility to participate in the study, and legal guardianship at the first home visit.

Inclusion criteria

- Family includes child with ASD, aged 3-16 years
- Diagnosis of autism spectrum disorder, severity level 1 or 2
- Diagnosed within 12 months of consenting to the study
- If other diagnoses are present, ASD must be primary diagnosis
- Family are willing to comply with study requirements

Exclusion criteria

- Children with ASD severity level 3
- Children with ASD and intellectual impairment*
- Serious concomitant illness in child or family, or other circumstances such that they are unable to comply with study requirements
- Families who may be a risk to safety of research staff (This will be assessed by the clinical and research staff on the basis of clinical records, diagnosis letter and contact prior to the first home visit).

For peer review only - http://bmjopen.bmj.com/site/about/guidelines.xhtml

- Insufficient English language or capacity for parent/child to consent/assent to the study.

* Intellectual impairment will be assessed by the clinical staff as present on the basis of any of the following criteria:

- The child has a comorbid diagnosis of intellectual disability
- Diagnosis specifies "with accompanying intellectual impairment"
- The child has been identified as requiring very substantial support (severity level 3) according to DSM-5 criteria for ASD
- The child is being educated in a special school for children with intellectual disabilities
- The child has an IQ of 70 or below

Study design

This is a randomised, controlled, multi-centred feasibility study including children with autism and their families. A total of 36 families will be recruited in four cohorts and each cohort will be randomised in a 2:1 ratio to receive support as usually employed (SUE) plus a programme of Systemic Autism-related Family Enabling (SAFE) therapy, or SUE alone, for a period of 16 weeks. Advantages of 2:1 allocation include:

- Increased appeal for patients deciding whether to consent to randomisation.
- Increased ability to test training of therapists, and ability to deliver high-fidelity treatment.
- Minimal reduction in statistical power for between-groups comparisons in a full-scale evaluation.
- Increased ability to recruit required number of families within an area before randomising; which will be closer to the figure needed if and when the intervention is implemented.

Outcome assessors will be blinded to allocation. All participants will complete outcome measures at baseline and again at 32 weeks post-allocation via a face-to-face visit, hence each family will participate in the SAFE study for approximately eight months. An embedded qualitative study will collect information about the feasibility and acceptability of the intervention and the study itself. Qualitative data will be collected at a Family Feedback Day after the 32 week post-allocation visits have been completed. The end date for the trial will be the date on which the last family completes the Family Feedback Day.

Outcome measures

Feasibility outcome measures:

- Ability to identify, recruit and randomise eligible families.

For peer review only - http://bmjopen.bmj.com/site/about/guidelines.xhtml

- Acceptability of proposed outcome measures and follow-up schedule to participants, and whether targets for loss to follow-up are achievable.
- Adherence of therapists and families to the intervention.
- Ability to gather quantitative data on outcomes
- Appropriateness of resource use questionnaires and preference-based instruments for this population
- Effectiveness and scalability of training arrangements

Clinical outcome measures:

- Scores on the proposed primary outcome measure, the Systemic CORE 15 (SCORE) [43]. This is a 15 item paper-based survey, which has been shown to have good internal reliability (Cronbach's α = .89) [44] and to be a valid index of family functioning, taking approximately 20 minutes to complete. The SCORE is the primary measure of family functioning employed in CYP (Children and Young People's) Improving Access to Psychological Therapies national programme, and is the gold standard for assessing the impact of family therapy on quality of life in the UK [45]. Every able family member will be asked to complete the SCORE, and the same family members should complete the SCORE at baseline and 32 weeks. The SCORE-15 is freely available online [46].

- Scores on the proposed secondary outcome measures, which index changes in child behaviour, child-parent attachment, anxiety, and depression.
    - Patient Health Questionnaire – Somatic Anxiety Depressive Symptoms (PHQ-SADS). This comprises the PHQ-9 (estimated internal reliability Cronbach's α = .86-.89) [47] measuring depression and the GAD-7 (estimated internal reliability α =.92 [47] measuring anxiety [48].
    - Adapted mutuality sub-scale of the Coding of Attachment-Related Parenting for use with children with Autism - CARP-A [49]. The CARP-A is a validated observational measure of a child with autism's attachment behaviour towards their carer The CARP-A Mutuality subscale is reported as having inter-rater reliability of .74 [50].
    - The Child Behaviour Checklist (CBCL) [51]. This is a 30-item paper-based survey, which detects emotional and behavioural problems. Reasonable internal reliability is reported for each of three scales, given that some scales only have 4 items: 1. Competence scales (Cronbach's α = .63-.79) 2. Problems scales (Cronbach's α = .78-.97) and 3. DSM orientated scales (Cronbach's α = .72-.91) [52].
    - The Reflective Functioning Questionnaire (RFQ) [53] measures ability to understand own and others' mental states (Test-retest reliability coefficients are reported as 0.84) [53].
    - Caregiving Helplessness Questionnaire [54] (CGHQ). This is a 26-item questionnaire designed to assess aspects of disorganised caregiving. The

For peer review only - http://bmjopen.bmj.com/site/about/guidelines.xhtml

CGHQ includes three subscales with reasonable internal reliability given the number of items: 1. Mother Helpless (α = .86) includes 7 items, 2. Mother-Child Frightened (α = .66) includes 6 items, 3. Child Caregiving (α = .64) includes 6 items [55].

- Scores on generic health economic outcome measures will also provide data on the feasibility of cost effectiveness analysis:
  - EuroQol 5 dimensions (EQ-5D-5L). This is a standardised generic instrument for measuring health outcome.
  - Child Health Utility 9 Dimensions (CHU-9D). The CHU-9D is a paediatric generic preference based measure of health related quality of life.
  - Resource Use Questionnaire (RUQ). A paper-based questionnaire completed by parent about his/her child's use of health care and social resources. The RUQ is designed to identify the NHS and Social Care resource use for the economic evaluation. It includes items to establish number and type of health resources being used, such as number of GP visits or number of days in hospital. Resource Use Questionnaire completion will be matched with medical records for a subgroup of families, which will help to develop strategies to minimise missing data in the future definitive trial.

- Qualitative outcomes:
  - Acceptability of SAFE and the trial process for participants and therapists
  - Reasons for declining and withdrawing from the study

The qualitative component will employ focus groups and interviews to investigate four key aspects of the study experience: families' experiences of the study (including intervention and potential harm of the intervention), therapists' experiences of the intervention, reasons for eligible families declining, and reasons for families withdrawing from the study. After the 32-week assessments have been completed, participating families that have consented to participate in the qualitative focus groups will be given details of the time and location for the family feedback day. The family feedback day will involve several separate focus group sessions organised to take place over the period of a morning or an afternoon at a local venue for each centre including focus groups aimed specifically at parents and at children.

The Intervention

SAFE is a manualised intensive programme of systemic family therapy designed to treat maladaptive autistic symptoms and mental health related difficulties encountered by families of children with autism. SAFE provides a toolkit of therapeutic activities based on Attachment Theory, established systemic practice and the known visual processing preferences of people with autism [For example, 56,57,58]. Each therapy session will include two therapists with a minimum of intermediate family therapy level of qualification

For peer review only - http://bmjopen.bmj.com/site/about/guidelines.xhtml

and four days training in SAFE principles. Prior to the therapy sessions parents allocated to SAFE will complete an adapted version of the Parent Development Interview [59], which will provide therapists with background information on family experience. The reflective functioning questions of this interview will also be revisited as an opportunity to discuss change at the 24-week follow up. Between weeks 1 and 16, families allocated to the SAFE intervention will attend five 3-hour SAFE therapy sessions. Sessions 1 and 5 are multi-family sessions and will take place in a community setting. Sessions 2, 3 and 4 are for individual families and will take place in a community venue or the family home. The therapists will facilitate sessions which will be video recorded, as is usual practice for therapy sessions. The videotapes will be used by the therapists in supervision sessions and preparation for subsequent sessions.

Following completion of the therapy programme, families will attend a group follow-up session at 24 weeks post-allocation. Families will discuss any changes they have encountered focusing on their ability to be reflective about challenges faced and solutions tried. Trained support workers from local voluntary groups will attend this follow-up session and will be invited to give the families information about continued support for families of children with autism through existing networks.

Each session will include the following assessments for families to complete:

1. Client Satisfaction Questionnaire (CSQ-8)
2. The Helpful Aspects of Therapy Questionnaire (HAT)
3. A Between Session Activity (BSA) homework activity Families will be encouraged to complete a pro-forma with key elements of the intervention as prompts for families to track strengths and difficulties in response to SAFE ideas. Completion of the BSA will be recorded.

At the end of each session the therapists will also complete a training checklist and questionnaire to monitor protocol adherence.

For peer review only

Support as usually employed

Families will typically be offered a post-diagnosis follow-up appointment with the diagnosing paediatrician. Parents of children whose symptoms are not severe may be directed to local authority parenting classes. Classes such as ‘Timeout for Autism’ (Cornwall and the Isles of Scilly) and ‘Understanding Autism’ (Plymouth) focus on the features of Autism Spectrum Disorder, instructional parenting techniques and issues associated with education. Psycho-education may also be offered, with families being directed to relevant resources for e.g. The National Autism Society, Gateway ASD, and the NHS Child and Adolescent Mental Health Services (CAMHS). For families where a member is experiencing depression or anxiety, treatment varies and is not linked to autism-related care. Initial referral is often through the GP. Patients may receive Cognitive Behavioural Therapy as part of the

For peer review only - http://bmjopen.bmj.com/site/about/guidelines.xhtml

Improved Access for Psychological Therapies service. They may also receive medication and in extreme cases a period of in-patient hospital treatment.

A study schema is presented in Figure 1. below.

(Place Figure 1 here)

Proposed sample size

In this feasibility trial no formal statistical testing of between group differences is planned. Sample size has been selected heuristically with the goal of i) demonstrating that participants can be recruited at a rate sufficient to run a full scale evaluation of SAFE at a later date; ii) demonstrating that it is possible to train therapists and deliver SAFE to patients within the study treatment settings, and iii) demonstrate that the data collection procedures are effective, and that the data collection is acceptable to the 36 families, and not overly burdensome.

Data analysis

Completed paper case report forms will be checked and signed by research staff before being sent to the Peninsula Clinical Trials Unit (CTU). Original case report form pages will be posted to the CTU at agreed time points for double-data entry on to a password-protected database, with copies retained at the study site. Forms will be tracked using a web-based trial management system. Data will be analysed and presented as is appropriate for a feasibility study, in particular concentrating on descriptive analyses and undertaking no formal comparisons between groups. Reporting will follow the principles of the CONSORT Statement using the checklist and flowchart as recommended in the CONSORT extension for Randomized Pilot and Feasibility [60]. The flowchart will provide detail about the number of families approached, number eligible, number consenting, number randomised, number receiving allocated intervention and number assessed for outcome data at each time point. As appropriate, details will be given for individual members of the family, for example, how many family members there are and how many completed each questionnaire. Wherever possible, detailed reasons will be given for exclusions, loss to follow-up, non-completion of outcome measures etc.

Numbers will also be provided by centre and group, to inform the logistics of recruiting nine families prior to randomisation and following them up after randomisation. For those randomised to the SAFE intervention, adherence will be reported according to the number of group sessions attended and participation of individual family members at each of the therapy sessions. Completeness of data will be reported for each outcome measure at each relevant time point. Again this will be reported for individual family members as appropriate.

For each outcome measure, the relevant scores will be calculated and presented descriptively by trial arm. Where available, published guidelines will be used to process,

For peer review only - http://bmjopen.bmj.com/site/about/guidelines.xhtml

score and summarise the measures including, for example, the use of imputation in the event of missing items on a questionnaire. Summary measures will be calculated as appropriate, for example, means and standard deviations, medians and ranges, numbers and percentages in categories. These measures will be presented both for baseline and for the final follow-up. The only analysis contrasting the two groups will be an interval estimate in the form of a 95% confidence interval for the primary outcome, so that the plausibility for the effect size used in the sample size calculation for the full trial can be assessed. For this purpose the baseline values will be used in an Analysis of Covariance, with acknowledgment that no effects are included for group or therapist.

Focus group interviews will be audio recorded and transcribed verbatim. Consequent qualitative data will be managed using proprietary computer assisted qualitative data analysis software, for example, Nvivo 10, and analysed thematically [61,62]. Rigour of analysis will include 'respondent validation', whereby participants are provided with a summary of their transcript and analysis so that they can assess whether the interpretations being made about the data, accurately represent them. In addition, a second qualitative researcher will conduct an independent analysis of a subset of half of the focus group transcripts. Researchers will then meet to discuss and agree the findings, which will then be presented to the Family Consultation Group for discussion.

Patient and Public Involvement

Families of children with autism initiated the development of this project by communicating their complex needs and dissatisfaction with current service provision through the Plymouth Autism Network. This Network was set up by the Chief Investigator in 2011 to bring clinicians, carers, academics and individuals with autism together to share ideas, research findings and experiences. We further explored the challenges facing families of children with autism by conducting in-depth interviews and surveying over 90 families regarding their needs and the treatment they received post-diagnosis. Less than 9% of families agreed that current treatment helped with the problems they face. Our survey revealed a strong need for interventions, which support the whole family.

This pilot data led to the development of a research team within the Welcome Research Hub at Plymouth University, which included a Family Consultation Group. Our Family Consultation Group worked with us to develop and refine the SAFE intervention prior to the current project. These families have also contributed to the creation of a recruitment and treatment plan, which will be manageable for families. They have offered advice about how it is best to communicate with families at the start of the study and as it progresses. In addition, the Family Consultation Group representative is a co-applicant on this study.

Our Family Consultation Group will continue to be essential members of the team and work as an advisory group throughout the feasibility study and beyond. We see our Family Consultation Group as experts in their own lives and the lives of families with similar

For peer review only - http://bmjopen.bmj.com/site/about/guidelines.xhtml

challenges. For this reason we feel our role is to work with them in a supportive manner as collaborators. Their contribution is valuable in the same way as other experts on the team and we aim to facilitate one another. As stated above, the Family Consultation Group have been active in contributing to the research plan. Their input is of particular value in developing recruitment procedures, designing participant information packs and providing information about potential barriers to retention. We have also worked with them to prepare and deliver a training programme for research staff and therapists. With their help we have trained recruited staff work in a sensitive and informed manner with participants. We also value their input in interpreting and reporting data; in particular commenting on possible ways to overcome challenges for the main RCT.

Our families can help by identifying local networks and sharing their experience with new groups. Our Family Consultation Group are proactive campaigners for change and have extensive knowledge of existing bodies such as the National Autistic Society.They can also provide a family-centred perspective on research outcomes. They are, therefore, well-placed to collaborate with us in planning next steps and disseminating findings at local and national levels.

ETHICS AND DISSEMINATION

Risks and safety

Families of children with autism are a vulnerable group. The risks associated with participating in this study are however, considered minimal, with no adverse events anticipated in any participant. For those in the intervention group, there is a slight chance that the SAFE family therapy sessions could lead to an initial increase in family disagreements as family members learn how to change the way they solve problems and talk with one another. However, the purpose of the intervention is ultimately to equip families with skills to handle these difficulties by learning how to change the way they solve problems and improve their communication, and the SAFE family therapists will be available to provide support and will be trained to handle any emerging problems. Should any issues arise the SAFE family therapists will have access to two consultant clinical psychologists to provide further support and advice.

During the trial the children with autism will remain in the care of the Child Development Centre or the Autism Spectrum Disorder Assessment Team and will have access to usual care should any unforeseen circumstances arise. Other members of the family will also continue to be able to seek care and advice from the GP or any other specialist services they are concurrently involved with.

Monitoring adverse events

For peer review only - http://bmjopen.bmj.com/site/about/guidelines.xhtml

The research team have mechanisms in place to report serious adverse events (SAE) related to mental health. Serious Adverse Events related to mental health may be volunteered by the participant or discovered by the therapists, research assistants or other member of the research team during the SAFE family therapy sessions, or as a result of direct reporting (e.g. by telephone) by a family member, independent clinician or other informant. Serious adverse events will be recorded from the time of consent until the date the participant completes the follow-up or withdraws from the study.

If the CI considers that the SAE is not, or is unlikely to be, associated with the trial, the CTU will obtain a second assessment of causality from an independent assessor. Any SAE which in the opinion of either adjudicator is possibly related to the trial will be reported to the Research Ethics Committee within 15 days of the local research team having become aware of the event. All SAEs will be followed until either stabilised if chronic conditions or resolved.

Dissemination

If the feasibility study meets progression criteria an important part of our dissemination plan is to raise awareness of the need for a larger multi-centre trial. We will, therefore, offer targeted summaries of our findings and presentations to policy makers. The findings will also be broadly disseminated, but in a manner appropriate to a feasibility study. We plan national conference presentations and published papers to inform clinicians, academics and therapists about the possible benefits of SAFE and generate interest in the future trial. We will make use of our existing connections including the Association for Family Therapy, the National Autistic Society and the Institute of Family Therapy to reach relevant audiences. Our qualitative findings will also be published with detailed accounts of the families' reactions to SAFE and their views on its effectiveness. We will also provide forums for participating families to share their own experiences of the intervention with wider audiences through existing networks, groups and events across the UK.

Informing potential participants of possible benefits and known risks

The participant information sheets and leaflets will provide potential participants with information about the possible benefits and risks of taking part in the trial. For example, the participants will be informed that a potential risk of receiving the SAFE therapy is that the sessions may evoke difficult emotions and feelings this could lead to family disagreement as they move towards change. The families will also be informed that benefits of the trial include the possibility of improved coping skills when faced with challenges and contribution to finding out if SAFE can progress to a national trial. Participants will be given the opportunity to discuss risks and benefits with a member of the research team prior to consenting to participate.

Obtaining Informed consent from participants

For peer review only - http://bmjopen.bmj.com/site/about/guidelines.xhtml

All participants will receive a leaflet and information sheet prior to consent. There are two versions of the information sheet, one for adults and one for children. In the leaflet parents are encouraged to explain the trial to their younger children and some guidance for doing this is provided. The information sheet states that the participants have the right to withdraw at any point during the trial and that data collected from them will be confidential. All participants will have a home-visit prior to consent from a member of the research team and will be able to ask questions or go through the information verbally. Participants will have the process of the study explained to them including the estimated time they will have to wait prior to randomisation and starting the intervention if allocated to that arm.

Data protection/confidentiality

Participants will be given a unique identification number. The data will be pseudo-anonymised in the sense that there will be an identification number on the documentation but otherwise no means of identifying the individual to which the data relates. The research team will ensure that participants' pseudo-anonymity is maintained on all documents. Data will be collected and stored in accordance with the current legal and regulatory documentation. Electronic study records will be stored in a SQL server database, stored on a restricted access, secure server maintained by Plymouth University. Data will be entered into the database via a bespoke web-based data entry system encrypted using SSL. Access to electronic data will be permission based, and at the discretion of the clinical trials unit data management team.

Anonymised paper-based study data will be stored in locked filing cabinets. Copies of study data retained at the lead study site will be securely stored for the duration of the study prior to archiving. Video data will be transported via encrypted memory sticks and will be transferred to a password-protected computer. The clinical trials unit data team will have access to study data, including identifiable data. Other members of the study team and the trials unit will have restricted access to pseudo-anonymised study data. Access will be granted to the Sponsor and host institution on request, to permit study-related monitoring, audits and inspections.

For peer review only

Research governance and the conduct of the trial

The trial will be conducted to protect the human rights and dignity of the participant as reflected in the Helsinki Declaration. An important factor in protecting the participants is ongoing consultation with the SAFE Family Consultation Group. A representative of this group is a member of the research team and is involved in decision-making processes. The research team including the family consultation group are proactive in minimising discomfort and risk for participants, respecting their wishes over science and society, respecting the right to withdraw and the need for families to have access to all relevant information.

For peer review only - http://bmjopen.bmj.com/site/about/guidelines.xhtml

The Chief Investigator will be responsible for the overall conduct of the study, keeping it to schedule and within budget. Working closely with the CTU she will be the focal contact for enquiries from both sites. The CTU will manage the study, liaise with sites, monitor recruitment, work with the Sponsor and report to Trial Management Group (TMG) meetings. The TMG will meet regularly throughout the feasibility study. A Trial Steering Committee (TSC) will have an overarching monitoring responsibility. The TSC is expected to meet three times during the study, but will be additionally convened at the chairman or Chief Investigator's request.

Dissemination plans

If the feasibility study demonstrates successful recruitment, data collection and an ability to deliver the intervention, an important part the dissemination plan is to raise awareness of the need for a larger multi-centre trial. Targeted summaries of the findings and presentations will be disseminated to policy makers. The findings will also be broadly disseminated, but in a manner appropriate to a feasibility study. National conference presentations and published papers will be prepared to inform clinicians, academics and therapists about our feasibility results and generate interest in the future trial. Existing connections including the Association for Family Therapy, the National Autistic Society and the Institute of Family Therapy will be utilised to reach relevant audiences. The qualitative findings will also be published with detailed accounts of the families' reactions to SAFE and their views on its usefulness. A summary of study results in plain English will be available on the Peninsula Clinical Trials website.

Clinical trials authorisation and ethical approval

Clinical trials authorisation is not required. The study has appropriate Research Ethics Committee (REC) approval from the South West-Exeter Research Ethics Committee (Ref: 17/SW/0192) and approval from the Health Research Authority (HRA). The trial will be conducted in accordance with the protocol, the principles of the Declaration of Helsinki and ICH GCP. Any amendments of the protocol will be submitted to the Sponsor, HRA and REC for approval.

Trial sponsorship

The trial is sponsored by University Hospitals Plymouth NHS Trust.

Trial Steering Committee

The Trial Steering Committee will include an independent chair and at two other independent members, along with the lead investigator and the other study collaborators including a parent representative. They will meet once a year.

For peer review only - http://bmjopen.bmj.com/site/about/guidelines.xhtml

REFERENCES

1. National Autistic Society, 'Autism Facts and History', accessed 22 March 2018

2. American Psychiatric Association. Diagnostic and Statistical Manual of Mental Disorders, 5th edn. American Psychiatric Association, Washington, DC 2013.

3. Sasson NJ, Lam KSL, Parlier M, et al. Autism and the broad autism phenotype: familial patterns and intergenerational transmission. *J Neurodev Disord* 2013; 5:11-18.

4. Dykens EM, Fisher MH, Taylor JL, et al. Reducing Distress in Mothers of Children with Autism and Other Disabilities: A Randomized Trial. *Pediatrics* 2014; 2013-3164.

5. Hallett V, Ronald A, Colvert, E, et al. Exploring anxiety symptoms in a large-scale twin study of children with autism spectrum disorders, their co-twins and controls. *Journal of Child Psychology and Psychiatry* 2013;54:1176-1185.

6. Brereton AV, Tonge BJ, Einfeld SL. Psychopathology in children and adolescents with autism compared to young people with intellectual disability. *J Autism Dev Disord* 2006;36: 863-870.

7. Kim JA, Szatmari P, Bryson SE, et al. The prevalence of anxiety and mood problems among children with autism and Asperger syndrome. *Autism* 2000; 4:117-132.

8. Howlin P. Interventions for people with autism: recent advances. *Adv Psychiatr Treat* 1997;3: 94-102.

9. Mazefsky CA, Folstein SE, Lainhart JE. Overrepresentation of Mood and Anxiety Disorders in Adults with Autism and Their First Degree Relatives: What Does it Mean? *Autism Res* 2008;1:193-197.

10. Daniels JL, Forssen U, Hultman M, et al. Parental Psychiatric Disorders Associated With Autism Spectrum Disorders in the Offspring. *Pediatrics* 2008;121:1357-1162.

11. Siklos S, Kerns KA. Assessing Need for Social Support in Parents of Children with Autism and Down Syndrome. *J Autism Dev Disord* 2008;36:921-933.

12. Markoulakis R, Fletcher P, Bryden P. Seeing the glass half full: benefits to the lived experiences of female primary caregivers of children with autism. *Clin Nurse Spec.* 2012;26(1):48–56.

For peer review only - http://bmjopen.bmj.com/site/about/guidelines.xhtml

13. Potter CA. 'I accept my son for who he is – he has incredible character and personality': fathers' positive experiences of parenting children with autism. *Disabil Soc,* 2016;31(7):948-965.

14. Myers BJ, Mackintosh VH, Goin-Kochel RP. "My greatest joy and my greatest heart ache:" Parents' own words on how having a child in the autism spectrum has affected their lives and their families' lives. *Res Autism Spectr Disord*. 2009;3(3):670-684.

15. Zhang W, Yan TT, Barriball KL, While AE, Liu XH. Post-traumatic growth in mothers of children with autism: a phenomenological study. *Autism.* 2015;19(1):29-37.

16. Altiere MJ, von Kluge S. Searching for acceptance: challenges encountered while raising a child with autism. *J Intellect Dev Disabil.* 2009;34(2):142-152.

17. McCrone P, Dhanasiri S, Patel A, et al. The cost of mental health care in England to 2026. Executive Summary, London: The King's Fund. 2008

18. Buescher AVS, Cidav Z, Knapp M, et al. Costs of Autism Spectrum Disorders in the United Kingdom and the United States. *JAMA Pediatr* 2014;e1-e8.

19. Osborne LA, McHugh L, Saunders J, et al. Parenting Stress Reduces the Effectiveness of Early Teaching Interventions for Autistic Spectrum Disorders. *J Autism Dev Disord* 2008;38:1092-1103.

20. Pisula E. Parenting Stress in Mothers and Fathers of Children with Autism Spectrum Disorders, A Comprehensive Book on Autism Spectrum Disorders, Dr. Mohammad-Reza Mohammadi (Ed.) 2011. Available from: http://www.intechopen.com/books/a-comprehensive- bookon- autism-spectrum-disorders/parenting-stress-in-mothers-and-fathers-of-children-with-autismspectrum-disorders

21. Bolton PF, Pickles A, Murphy M, et al. Autism, affective and other psychiatric disorders: patterns of familial aggregation. *Psychological Medicine* 1998;28: 385-395.

22. Demir T, Dogangun B, Karacetin G, et al. Psychiatric Disorders and Symptoms in Mothers of Children with Autistic Disorder. *Neurol Psychiatry Brain Res* 2008;15:1-5.

23. Piven J, Palmer P. Psychiatric disorder and the broad autism phenotype: evidence from a family study of multiple-incidence autism families. *Am J Psychiatry* 1999;156:557-563.

24. Nikmat AW, Ahmad M, Oon NL, et al. Stress and psychological wellbeing among parents of children with autism spectrum disorder. *ASEAN Journal of Psychiatry* 2008;9:65-72.

For peer review only - http://bmjopen.bmj.com/site/about/guidelines.xhtml

25. Roberts AL, Koenen KC, Lyall K, Ascherio A, Weisskopf MG. Women's posttraumatic stress symptoms and autism spectrum disorder in their children. *Res Autism Spectr Disord* 2014;8(6):608-616.

26. Roberts AL, Lyall K, Rich-Edwards JW, Ascherio A, Weisskopf MG. Maternal exposure to intimate partner abuse before birth is associated with autism spectrum disorder in offspring. *Autism* 2016;20(1):26-36.

27. Konstantareas MK. Autistic, learning disabled and delayed children's impact on their parents. *Can J Behav Sci* 1991;23:358-375.

28. Murphy M, Bolton PF, Pickles A, et al. Personality traits of the relatives of autistic probands. *Psychol Med* 2000;30:1411-1424.

29. Blackledge JT, Hayes SC. Using Acceptance and Commitment Training in the Support of Parents of Children Diagnosed with Autism. *Child & Family Behavior Therapy,* 2006:28:1-18.

30. NICE guidelines [CG170] https://www.nice.org.uk/guidance/cg170/chapter/patient-centred-care

31. NICE guidelines [CG128] https://www.nice.org.uk/guidance/cg128

32. Munroe E. The Munroe Review of Child protection: Final report, Dept. of Education, HM Stationary Office, UK 2011.

33. Goodman S, Trowler I. Social work re-claimed: Innovative frameworks for child and social work Practice. London: Jessica Kingsley 2012.

34. NHS England. Future in Mind: promoting, protecting and improving our children and young people's mental health and wellbeing. Dept of Health Publication Gateway Ref. No 02939, 2015.

35. IAPT | CYP IAPT Children and Young People's Project. (n.d.). Retrieved January 5, 2015, from http://www.iapt.nhs.uk/cyp-iapt/

36. Bromley J, Hare DJ, Davison K. et al. Mothers supporting children with autistic spectrum disorders: Social support, mental health status and satisfaction with services. *Autism* 2004;8:409-423.

37. Stratton P. Report On The Evidence Base Of Systemic Family Therapy. *Association for Family Therapy* 2005.

38. Dallos R, Draper R. (4th Edn) An Introduction to Family Therapy. Maidenhead: Open Univ. Press/McGraw Hill 2015.

For peer review only - http://bmjopen.bmj.com/site/about/guidelines.xhtml

39. Monteiro M. Family Therapy and the Autism Spectrum: Autism Conversations in Narrative Practice. London: Routledge 2016.

40. Estrada AU, Pinsof WF. The effectiveness of family therapies for selected behavioural disorders of childhood. *J Marital Fam Ther* 1995;21: 403-424.

41. Pellicano E, Dinsmore A, Charman T. What should autism research focus upon? Community views and priorities from the United Kingdom. *Autism, Special Issue Article* 2014:1-15.

42. Wallace S, Parr J, Hardy, A. One in a hundred: Putting families at the heart of autism research. London: Autistica 2013.

43. Stratton P, Bland J, Janes E, et al. Developing an indicator of family function and a practicable outcome measure for systemic family and couple therapy: the SCORE. *Journal of Family Therapy* 2010;32:232-258.

44. Stratton P, Lask J, Bland J, Nowotny E, Evans C, Singh R, Janes E, Peppiatt A. Detecting therapeutic improvement early in therapy: validation of the SCORE-15 index of family functioning and change. *J Fam Ther* 2014;36:3-19.

45. SHIFT Self-Harm Intervention, Family Therapy: a randomised controlled trial of family therapy vs. treatment as usual for young people seen after second or subsequent episodes of self harm. Protocol Grant Number: 470772 HTA Reference Number: 07/33/01, 2015.

46. SCORE-15 [Internet] Association for Family Therapy www.aft.org.uk. [cited 2018 Sept 17] Available from: http://www.aft.org.uk/SpringboardWebApp/userfiles/aft/file/SCORE%20info/SCORE-15%20102013.pdf

47. Kroenke K, Spitzer RL, Williams JBW, Lowe B. The Patient Health Questionnaire Somatic, Anxiety, and Depressive Symptom Scales: a systematic review. *Gen Hosp Psychiatry* 2010;32:345-359.

48. Spitzer R L, Kroenke K, Williams JBW. The Patient Health Questionnaire Primary Care Study Group. Validation and utility of a self-report version of PRIME-MD: the PHQ Primary Care Study. *JAMA,* 282, 1999:1737-1744.

49. Blazey L. Coding of Attachment-Related Parenting for use with children with Autism (CARP-A) [dissertation]. Manchester: Manchester University, 2007.

50. Holt CE. Child-caregiver Play and Attachment in Pre-school Autism: A Longitudinal Investigation [thesis]. Manchester: University of Manchester, 2011.

51. Moss E, Bureau J, Cyr C, et al. Correlates of attachment at age 3 Construct validity of the Preschool Attachment Classification System. *Dev. Psychol* 2004;40:323-334.

For peer review only - http://bmjopen.bmj.com/site/about/guidelines.xhtml

52. Achenbach TM, Rescorla LA. Manual for the ASEBA School-Age Forms and Profiles: Child Behaviour Checklist for Ages 6-18. Burlington, VT: University of Vermont, Research Center for Children, Youth, & Families. 2001.

53. Fonagy P, Luyten P, Moulton-Perkins A, et al. Development and validation of a self-report measure of mentalizing: The Reflective Functioning Questionnaire. PLOS ONE. 2016;11(7):e0158678.

54. Solomon J, George C. (Eds.) Disorganised Attachment and Caregiving. Guilford: New York; 2011.

55. Huth-Bocks AC, Guyon-Harris K, Calvert M, Scott S, Ahlfs-Dunn S. The Caregiving Helplessness Questionnaire: Evidence for validity and utility with mothers of infants. *Infant Ment Health J.* 2016;37:208-221.

56. Ogletree BT, Morrow-Odom KL, Wrestling D. Understanding the brain–behaviour relationship in persons with ASD: Implications for PECS as a treatment choice. *Dev Neurorehabil* 2015;18(2): 88-96.

57. Buron KD, Wolfberg P. Learners on the autism spectrum: Preparing highly qualified educators (pp. 114–137). AAPC Publishing: Shawnee; 2008.

58. Grandin T, Scariano MM. (1986). Emergence: Labeled autistic. New York, NY: Warner Books 1986.

59. Slade A, Aber JL, Bresgi I, Berger B, Kaplan M. The parent development interview, revised. Unpublished protocol. The City University of New York 2004.

60. CONSORT extension for Randomized Pilot and Feasibility Trials (http://www.consort-statement.org/randomised-pilot-and-feasibilitytrials).

61. Braun V, Clarke V. Using thematic analysis in psychology. *Qual Res Psychol.* 2006;3:77-101.

62. Green J, Thorogood N. Qualitative Health Methods for health Research. London: Sage Publications; 2004.

AUTHORS' CONTRIBUTIONS: RM, JV and HH were responsible for the overall development of the protocol. RM, HH, RD, CM, PE, AB, TV and the Family Consultation Group were involved in the conception and production of the study and the development of the initial protocol. PE and AB provided methodological expertise and advice on quantitative analysis PE provided statistical expertise. RD was the lead researcher on design of the intervention and the qualitative component. TV, with the support of the Family Consultation Group, and CM advised on design and ethics, particularly from the participant perspective.

For peer review only - http://bmjopen.bmj.com/site/about/guidelines.xhtml

All authors made substantial contributions to drafting, revision and approval of the document.

ACKNOWLEDGEMENTS: The Authors would like to thank the SAFE Family Consultation Group for their ongoing input and expertise. They would also like to thank James Cook, Mary Hosken, Dr Ben Whalley, Dr Antonieta Medina-Lara and Professor Julian Archer for their help in developing and conducting this study.

FUNDING STATEMENT: This work was supported by the National Institute for Health Research (NIHR) grant number: PB-PG-0815-20058

COMPETING INTERESTS STATEMENT: Contributors are co-applicants or employed research staff on the SAFE project, which receives funds from both NIHR and Autistica. Professor Rudi Dallos holds joint Intellectual Property rights for the SAFE intervention with The University of Plymouth. No other issues were raised with regard to competing interests.

PROVENANCE AND PEER REVIEW: Not commissioned; externally peer reviewed.

DATA SHARING STATEMENT: Further details of the study protocol can be requested from the corresponding author.

Legend for Figure 1.

Figure 1. SAFE study schema

For peer review only

For peer review only - http://bmjopen.bmj.com/site/about/guidelines.xhtml

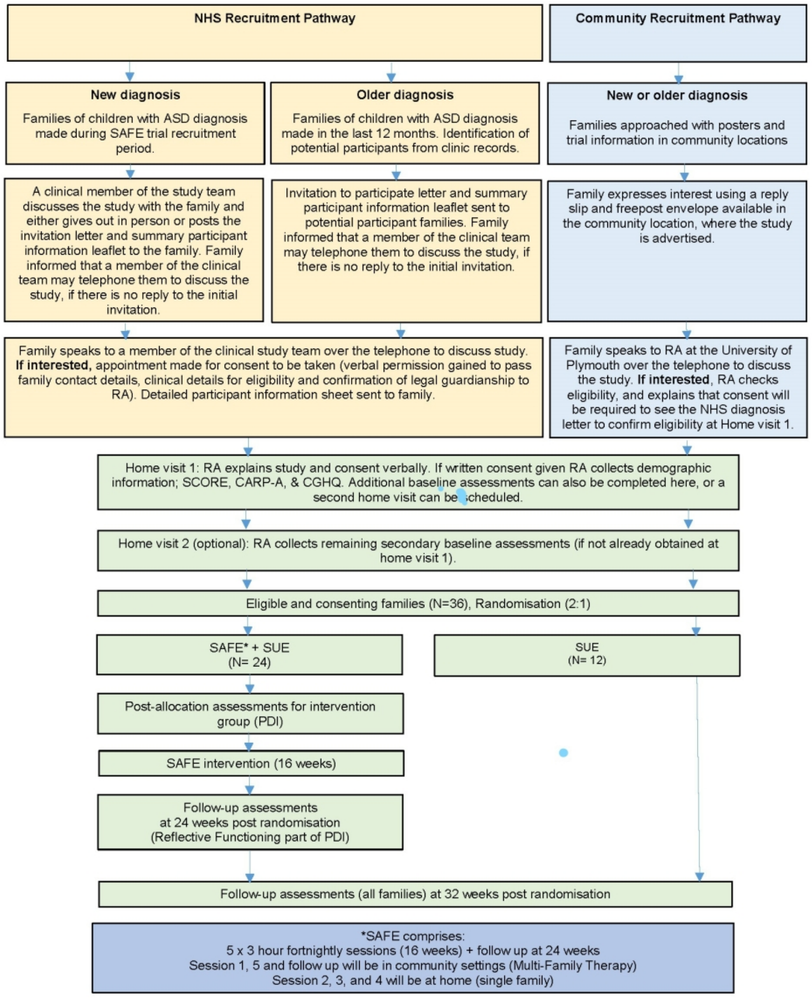
NHS Recruitment Pathway
Community Recruitment Pathway
New diagnosis
Families of children with ASD diagnosis made during SAFE trial recruitment period.
Older diagnosis
Families of children with ASD diagnosis made in the last 12 months. Identification of potential participants from clinic records.
New or older diagnosis
Families approached with posters and trial information in community locations
A clinical member of the study team discusses the study with the family and either gives out in person or posts the invitation letter and summary participant information leaflet to the family. Family informed that a member of the clinical team may telephone them to discuss the study, if there is no reply to the initial invitation.
Invitation to participate letter and summary participant information leaflet sent to potential participant families. Family informed that a member of the clinical team may telephone them to discuss the study, if there is no reply to the initial invitation.
Family expresses interest using a reply slip and freepost envelope available in the community location, where the study is advertised.
Family speaks to a member of the clinical study team over the telephone to discuss study. If interested, appointment made for consent to be taken (verbal permission gained to pass family contact details, clinical details for eligibility and confirmation of legal guardianship to RA). Detailed participant information sheet sent to family.
Family speaks to RA at the University of Plymouth over the telephone to discuss the study. If interested, RA checks eligibility, and explains that consent will be required to see the NHS diagnosis letter to confirm eligibility at Home visit 1.
Home visit 1: RA explains study and consent verbally. If written consent given RA collects demographic information; SCORE, CARP-A, & CGHQ. Additional baseline assessments can also be completed here, or a second home visit can be scheduled.
Home visit 2 (optional): RA collects remaining secondary baseline assessments (if not already obtained at home visit 1).
Eligible and consenting families (N=36), Randomisation (2:1)
SAFE* + SUE (N= 24)
SUE (N= 12)
Post-allocation assessments for intervention group (PDI)
SAFE intervention (16 weeks)
Follow-up assessments at 24 weeks post randomisation (Reflective Functioning part of PDI)
Follow-up assessments (all families) at 32 weeks post randomisation
*SAFE comprises:
5 x 3 hour fortnightly sessions (16 weeks) + follow up at 24 weeks
Session 1, 5 and follow up will be in community settings (Multi-Family Therapy)
Session 2, 3, and 4 will be at home (single family)

STANDARD PROTOCOL ITEMS: RECOMMENDATIONS FOR INTERVENTIONAL TRIALS

SPIRIT 2013 Checklist: Recommended items to address in a clinical trial protocol and related documents*

Section/item	Item No	Description	Addressed on page number
**Administrative information**			
Title	1	Descriptive title identifying the study design, population, interventions, and, if applicable, trial acronym	Front page
Trial registration	2a	Trial identifier and registry name. If not yet registered, name of intended registry	Front page
	2b	All items from the World Health Organization Trial Registration Data Set	Front page, 6, 9
Protocol version	3	Date and version identifier	Front page
Funding	4	Sources and types of financial, material, and other support	34
Roles and responsibilities	5a	Names, affiliations, and roles of protocol contributors	6
	5b	Name and contact information for the trial sponsor	6
	5c	Role of study sponsor and funders, if any, in study design; collection, management, analysis, and interpretation of data; writing of the report; and the decision to submit the report for publication, including whether they will have ultimate authority over any of these activities	5
	5d	Composition, roles, and responsibilities of the coordinating centre, steering committee, endpoint adjudication committee, data management team, and other individuals or groups overseeing the trial, if applicable (see Item 21a for data monitoring committee)	30 - 32

For peer review only - http://bmjopen.bmj.com/site/about/guidelines.xhtml

**Introduction**			
Background and rationale	6a	Description of research question and justification for undertaking the trial, including summary of relevant studies (published and unpublished) examining benefits and harms for each intervention	10 -11
	6b	Explanation for choice of comparators	29 - 30
Objectives	7	Specific objectives or hypotheses	11 - 12
Trial design	8	Description of trial design including type of trial (eg, parallel group, crossover, factorial, single group), allocation ratio, and framework (eg, superiority, equivalence, noninferiority, exploratory)	12
**Methods: Participants, interventions, and outcomes**			
Study setting	9	Description of study settings (eg, community clinic, academic hospital) and list of countries where data will be collected. Reference to where list of study sites can be obtained	16
Eligibility criteria	10	Inclusion and exclusion criteria for participants. If applicable, eligibility criteria for study centres and individuals who will perform the interventions (eg, surgeons, psychotherapists)	16
Interventions	11a	Interventions for each group with sufficient detail to allow replication, including how and when they will be administered	21 - 22
	11b	Criteria for discontinuing or modifying allocated interventions for a given trial participant (eg, drug dose change in response to harms, participant request, or improving/worsening disease)	21
	11c	Strategies to improve adherence to intervention protocols, and any procedures for monitoring adherence (eg, drug tablet return, laboratory tests)	21, 30
	11d	Relevant concomitant care and interventions that are permitted or prohibited during the trial	22
Outcomes	12	Primary, secondary, and other outcomes, including the specific measurement variable (eg, systolic blood pressure), analysis metric (eg, change from baseline, final value, time to event), method of aggregation (eg, median, proportion), and time point for each outcome. Explanation of the clinical relevance of chosen efficacy and harm outcomes is strongly recommended	14 - 15
Participant timeline	13	Time schedule of enrolment, interventions (including any run-ins and washouts), assessments, and visits for participants. A schematic diagram is highly recommended (see Figure)	19

For peer review only - http://bmjopen.bmj.com/site/about/guidelines.xhtml

Sample size	14	Estimated number of participants needed to achieve study objectives and how it was determined, including clinical and statistical assumptions supporting any sample size calculations	29 - 30
Recruitment	15	Strategies for achieving adequate participant enrolment to reach target sample size	20
**Methods: Assignment of interventions (for controlled trials)**			
Allocation:			
Sequence generation	16a	Method of generating the allocation sequence (eg, computer-generated random numbers), and list of any factors for stratification. To reduce predictability of a random sequence, details of any planned restriction (eg, blocking) should be provided in a separate document that is unavailable to those who enrol participants or assign interventions	29 - 30
Allocation concealment mechanism	16b	Mechanism of implementing the allocation sequence (eg, central telephone; sequentially numbered, opaque, sealed envelopes), describing any steps to conceal the sequence until interventions are assigned	19 - 20
Implementation	16c	Who will generate the allocation sequence, who will enrol participants, and who will assign participants to interventions	20
Blinding (masking)	17a	Who will be blinded after assignment to interventions (eg, trial participants, care providers, outcome assessors, data analysts), and how	20 - 21
	17b	If blinded, circumstances under which unblinding is permissible, and procedure for revealing a participant's allocated intervention during the trial	20 - 21
**Methods: Data collection, management, and analysis**			
Data collection methods	18a	Plans for assessment and collection of outcome, baseline, and other trial data, including any related processes to promote data quality (eg, duplicate measurements, training of assessors) and a description of study instruments (eg, questionnaires, laboratory tests) along with their reliability and validity, if known. Reference to where data collection forms can be found, if not in the protocol	27 - 29
	18b	Plans to promote participant retention and complete follow-up, including list of any outcome data to be collected for participants who discontinue or deviate from intervention protocols	24 - 25

For peer review only - http://bmjopen.bmj.com/site/about/guidelines.xhtml

Data management	19	Plans for data entry, coding, security, and storage, including any related processes to promote data quality (eg, double data entry; range checks for data values). Reference to where details of data management procedures can be found, if not in the protocol	28
Statistical methods	20a	Statistical methods for analysing primary and secondary outcomes. Reference to where other details of the statistical analysis plan can be found, if not in the protocol	29 - 30
	20b	Methods for any additional analyses (eg, subgroup and adjusted analyses)	25 - 26
	20c	Definition of analysis population relating to protocol non-adherence (eg, as randomised analysis), and any statistical methods to handle missing data (eg, multiple imputation)	29 - 30
**Methods: Monitoring**			
Data monitoring	21a	Composition of data monitoring committee (DMC); summary of its role and reporting structure; statement of whether it is independent from the sponsor and competing interests; and reference to where further details about its charter can be found, if not in the protocol. Alternatively, an explanation of why a DMC is not needed	31 - TSC performing DMC role.
	21b	Description of any interim analyses and stopping guidelines, including who will have access to these interim results and make the final decision to terminate the trial	33
Harms	22	Plans for collecting, assessing, reporting, and managing solicited and spontaneously reported adverse events and other unintended effects of trial interventions or trial conduct	26 - 27
Auditing	23	Frequency and procedures for auditing trial conduct, if any, and whether the process will be independent from investigators and the sponsor	30 - 31
**Ethics and dissemination**			
Research ethics approval	24	Plans for seeking research ethics committee/institutional review board (REC/IRB) approval	32 - 33
Protocol amendments	25	Plans for communicating important protocol modifications (eg, changes to eligibility criteria, outcomes, analyses) to relevant parties (eg, investigators, REC/IRBs, trial participants, trial registries, journals, regulators)	32

For peer review only - http://bmjopen.bmj.com/site/about/guidelines.xhtml

Consent or assent	26a	Who will obtain informed consent or assent from potential trial participants or authorised surrogates, and how (see Item 32)	18
	26b	Additional consent provisions for collection and use of participant data and biological specimens in ancillary studies, if applicable	23
Confidentiality	27	How personal information about potential and enrolled participants will be collected, shared, and maintained in order to protect confidentiality before, during, and after the trial	28 - 29
Declaration of interests	28	Financial and other competing interests for principal investigators for the overall trial and each study site	______________
Access to data	29	Statement of who will have access to the final trial dataset, and disclosure of contractual agreements that limit such access for investigators	29
Ancillary and post-trial care	30	Provisions, if any, for ancillary and post-trial care, and for compensation to those who suffer harm from trial participation	33
Dissemination policy	31a	Plans for investigators and sponsor to communicate trial results to participants, healthcare professionals, the public, and other relevant groups (eg, via publication, reporting in results databases, or other data sharing arrangements), including any publication restrictions	33 - 34
	31b	Authorship eligibility guidelines and any intended use of professional writers	______________
	31c	Plans, if any, for granting public access to the full protocol, participant-level dataset, and statistical code	______________
**Appendices**			
Informed consent materials	32	Model consent form and other related documentation given to participants and authorised surrogates	______________
Biological specimens	33	Plans for collection, laboratory evaluation, and storage of biological specimens for genetic or molecular analysis in the current trial and for future use in ancillary studies, if applicable	N/A

*It is strongly recommended that this checklist be read in conjunction with the SPIRIT 2013 Explanation & Elaboration for important clarification on the items. Amendments to the protocol should be tracked and dated. The SPIRIT checklist is copyrighted by the SPIRIT Group under the Creative Commons "Attribution-NonCommercial-NoDerivs 3.0 Unported" license.

For peer review only - http://bmjopen.bmj.com/site/about/guidelines.xhtml

BMJ Open

SAFE, a new therapeutic intervention for families of children with autism: study protocol for a feasibility randomised controlled trial

Journal:	*BMJ Open*
Manuscript ID	bmjopen-2018-025006.R2
Article Type:	Protocol
Date Submitted by the Author:	07-Mar-2019

Complete List of Authors:	McKenzie, Rebecca; University of Plymouth, Institute of Education<br>Dallos, Rudi; Department of Clinical Psychology, University of Plymouth<br>Stedmon, Jacqui; The University of Plymouth, Clinical Psychology<br>Hancocks, Helen<br>Vickery, Patricia; Plymouth University, Peninsula Clinical Trials Unit<br>Ewings, Paul; Research Design Service, Research Office<br>Barton, Andy; South West Research Design Service<br>Vassallo, Tara<br>Myhill, Craig

**Primary Subject Heading**:	Mental health
Secondary Subject Heading:	Public health, Pathology, Research methods
Keywords:	autism, family therapy, intervention, feasibility

SCHOLARONE™
Manuscripts

SAFE, a new therapeutic intervention for families of children with autism: study protocol for a feasibility randomised controlled trial

Rebecca McKenzie[1], Rudi Dallos[1], Jacqui Stedmon[1], Helen Hancocks[2], Jane Vickery[2], Paul Ewings[3], Andy Barton[3], Tara Vassallo[1], Craig Myhill[1]

Author affiliations

1. Plymouth University. Plymouth UK
2. Peninsula Clinical Trials Unit, Plymouth University. Plymouth UK
3. NIHR Research Design Service. South West. UK

Correspondence to: Dr Rebecca McKenzie rebecca.mckenzie@plymouth.ac.uk

Key words: Family Therapy, autism, intervention, feasibility, mental health

Word count: 5903 excluding title page, abstract and references

For peer review only

ABSTRACT

Introduction: Incidence of autistic traits, mental health problems, stress and poor coping is high among family members of children with autism. These problems are coupled with challenging behaviour among children with autism. Current treatment for these families is disjointed and costly. The need for whole family support is supported by NICE recommendations, developments regarding children's service provision, research and requests by families of children with autism. Despite evidence that family therapies can provide benefits to these families, its efficacy has not been subject to a randomised controlled trial. SAFE is a new family therapy intervention designed specifically for families of children with autism. We aim to establish the feasibility of running a fully powered randomised controlled trial to evaluate SAFE.

Methods and analysis: Families of children with autism aged 3-16 years will be invited to participate. Consenting participants will be randomised 2:1 to either SAFE + support as usual or support as usual alone. The proposed primary outcome measure for the main trial will be the Systemic CORE 15. Participants will also complete proposed secondary outcome measures, indexing changes in child behaviour, child-parent attachment, anxiety, and depression. Generic health economic outcome measures (EQ-5D-5L and CHU-9D) will also provide data on the feasibility of cost effectiveness analysis. Questionnaires will be completed at baseline and 32 weeks post allocation. Data on ability to identify, recruit, randomise, retain and collect data from families, acceptability of outcome measures, adherence of therapists and families to the intervention, appropriateness of resource use questionnaires and effectiveness of training will be collected for feasibility analysis. Qualitative data will also explore acceptability of SAFE and reasons for declining and withdrawing from the study.

Ethics and dissemination: The current trial protocol received ethical approval from the South West-Exeter Research Ethics Committee (Ref: 17/SW/0192). The findings of the trial

review only

For peer review only - http://bmjopen.bmj.com/site/about/guidelines.xhtml

will be disseminated in collaboration with our Family Consultation Group and other partners. Findings will be shared locally, nationally and internationally though events, conferences and published papers.

Trial registration number: ISCTRN83964946 IRAS 213527

For peer review

STRENGTHS AND LIMITATIONS OF THIS STUDY

- The study addresses a gap in the available research data, and will produce important feasibility information to inform a fully powered randomised controlled trial.
- The study explores the feasibility of using measures of family function and a range of mental health measures.
- Quantitative feasibility data are complemented by qualitative focus groups and interviews.
- The study explores the feasibility of economic analysis measures in a population, which includes adults and their children with developmental disorders.
- The participants are recruited from two NHS Trusts in adjacent counties in the South West of England, leading to potential bias. A future randomised controlled trial will extend to centres across the UK including Scotland and Wales.

For peer review only - http://bmjopen.bmj.com/site/about/guidelines.xhtml

INTRODUCTION

More than 1% of the UK population has a diagnosis of autism [1]. Families of children with autism present complex needs. Children with autism have impairments in social interaction, communication, and behavioural flexibility [2]. Autism is widely accepted to have a genetic component and the Broad Autism Phenotype is disproportionally represented among family members [3]. Mental health problems are experienced by more than 70% of individuals with autism and more than 50% of their parents [4,5]. Individuals with autism suffer from high levels of anxiety and depression compared to those with other developmental conditions [6,7,8]. Families of children with autism have higher rates of depression, anxiety, and social phobia, than families with typically developing children, or children with other developmental disorders [9]. Parents of children with autism are more likely to be hospitalised for mental disorders than parents of typically developing children [10]; and mothers of children with autism are reported to have higher unmet needs, more difficulties coping, and lower satisfaction with service interactions than mothers of children with other disabilities [11]. Aside from these reported difficulties, families of children with autism can have positive family experiences, sense of wellbeing [12] and positive perceptions of their children [13]. Despite challenges, autism can be seen as enhancing family experience and some parents recognise that parenting a child with autism has added joy to their lives [14], made them more appreciative [15], more patient and compassionate [16].

As families of children with autism often exhibit psychological morbidities alongside autism, costs of services to treat these problems are high [17,18]. Furthermore, untreated or unresponsive mental health problems impose societal costs making it hard for parents to interact effectively with services [19], potentially worsening outcomes for children and exacerbating the substantial economic burden of autism [18].

For peer review only - http://bmjopen.bmj.com/site/about/guidelines.xhtml

Explanations for high levels of affective disorders in these families include: stress associated with the condition of autism, genetic factors, and intergenerational family dynamics. Parenting children with autism involves stresses associated with challenging behaviour, lack of Theory of Mind, and atypical attachment behaviour displayed by children [20]. Parents of children with autism report that a consequent lack of psychological wellbeing exacerbates maladaptive behaviour in their children [21], which is likely to result in unhelpful cycles of distress and hopelessness.

Studies exploring the medical histories of family members indicate, that the onset of affective disorders may predate the birth of the child [9,22,23] suggesting that mental health difficulties cannot be wholly accounted for by stress involved in parenting. It seems, therefore, that these individuals may have been living with psychological distress for a long period of time. Depression and anxiety among family members have been tentatively linked to genetic factors independent of the Broad Autism Phenotype [24]. But few studies explore the intergenerational presence of affective disorder associated with autism [9,23,23].

Previous research demonstrates that experience of trauma and abuse among women is associated with elevated risk of autism developing in their subsequent offspring [25,26]. Hence mothers of children with autism are more likely than the general population to be coping with previous traumatic events. In addition, these families often encounter difficulties communicating needs to external agencies [27], which may trigger existing tendencies for negative affect. Families of children with autism can experience positive family life, cope well with difficulties and enjoy good relationships with their children, but they represent a high-risk group, for whom treatment is disjointed, costly and inadequate [28,29].

A more joined-up approach is required which focuses on autism related need, coping with challenging behaviour and mental health difficulties by encouraging fundamental reflective functioning and improving family dynamics. The SAFE study should be placed in the context of NICE guidelines and recommendations [30,31] as well as developments

For peer review only - http://bmjopen.bmj.com/site/about/guidelines.xhtml

regarding children's service provision proposed by the Munroe Report [32,33], and the 'Future in Mind' children and young people's mental health report [34]. The SAFE study also reflects recommendations by other researchers working in the field [35,36]. Families of children with autism themselves highlight the importance of professionals working therapeutically with children and the wider family, in contrast to parents of children with conditions such as Down Syndrome who tend to stress the support needs of their child within educational and community settings [10].

SAFE is a systemic family therapy approach designed by experts to address autism related needs including mental health difficulties and problematic behaviour. Systemic Family Therapy is a well-recognized, evidence-based psychotherapeutic approach [37], which is recommended treatment for conditions such as Conduct Disorder, ADHD and Anorexia Nervosa [38]. Despite evidence that family therapies can provide benefits to children with autism and their parents [39,40] its efficacy for treating this condition has not been subject to a randomised controlled trial. A comprehensive search of clinical trial registries revealed no on-going trials assessing Systemic Family Therapy as a treatment for autism and associated mental health problems. This is surprising given guidelines and recommendations for care; the successful use of family therapies for a range of conditions and reports documenting key areas of concern for the UK autism community [41,42], which overwhelmingly show that families of children with autism want interventions which make real improvements to their daily life and sense of wellbeing. Consequently, the overarching aim of this study is to establish the feasibility of a definitive randomised controlled trial to evaluate Systemic Autism-related Family Enabling (SAFE) therapy for families of children with autism.

METHODS AND ANALYSIS

Participants and recruitment

For peer review only - http://bmjopen.bmj.com/site/about/guidelines.xhtml

Our target population are families of children with autism, who do not have an intellectual impairment, between the ages of 3 and 16 years. SAFE is designed to have a visual, playful approach which draws from established principles of family therapy, where therapists and families work as collaborators to solve problems and effect change. SAFE activities are adaptable, family led and can be used flexibly according to the needs of the family and the age of the child. Children gain most from the intervention, however, if they can understand and communicate their responses to SAFE activities. Pilot data suggest that SAFE will be most effective and accessible for children who do not have severe symptoms or an intellectual impairment. Those children who were non-verbal and/or had severe communication difficulties found it difficult to engage with some activities. For this feasibility study, therefore, our target population is families of children with autism severity level 1 or 2 with no intellectual impairment. The authors are aware that high severity levels may not in all cases exclude children from engaging with SAFE and that the relationship between IQ and severity is complex. These issues will be explored as part of the feasibility outcomes, namely our ability to recruit eligible families.

Future plans for SAFE include the development of a sister intervention which has extended non-verbal elements based on Intensive Interaction and is designed specifically to support families of children with autism and an intellectual impairment. This feasibility study focuses on families of children of school-age which fits with the priorities of one of our secondary sources of funding. Background research exploring diagnostic data for our proposed centres for the previous two years revealed no children without intellectual impairment diagnosed before the age of 3 years. This information strongly suggested that we would be unable to recruit any families with children below the age of 3 years. Consequently, we focused on the 3-16 age group.

Participants will be identified and recruited from two study research sites: University Hospitals Plymouth NHS Trust (PHNT) Child Development Centre, and Cornwall Partnership NHS Foundation Trust Autism Spectrum Disorder Assessment Team (ASDAT). The pathways used to identify and recruit families will vary according to local practice, and the needs of the

For peer review only - http://bmjopen.bmj.com/site/about/guidelines.xhtml

individual families being approached. Some families will receive a diagnosis during the SAFE recruitment period, and others will have been diagnosed up to 12 months before the SAFE study recruitment period starts.

Families with a diagnosis during the SAFE recruitment period will be approached by the diagnosing paediatrician, who will perform an initial eligibility check, invite the families to find out more and, if interested, refer the family to a member of the local SAFE study team. Families with a diagnosis before the SAFE study recruitment period will be identified as potentially eligible from clinic records by a suitably qualified member of the clinical team at each centre. Clinical staff in our centres and the surrounding areas are responsible for diagnosis of the children within our participating families. If the child is recruited from a diagnostic centre the clinical staff also assess eligibility. The severity levels of the children and their intellectual ability are assessed on the autism pathways in Plymouth and Cornwall by a multi-disciplinary clinical team including educational and clinical psychologists, speech and language therapists and paediatricians. Assessment on the pathways occurs over a period of several months. This includes measures of IQ based on the Wechsler Intelligence Scale for Children WISC-V [43] and measures of intellectual functioning based on the British Ability Scales BAS3 [44] as well as observations and detailed reports from the schools or nursery settings and the family.

All potential participant families will receive a participant information leaflet including an invitation to take part. All interested families will be able to speak to a member of the study team to discuss the study and have any questions answered. The participant information leaflet will contain information about the study in plain English. Parents will be asked to explain the information to younger children in a way that is appropriate for their child and suggestions for how to do this will be contained in the leaflet. A home visit will be arranged by a member of the study team for those families who express interest in participating. During the visit, a research assistant will provide the families with more detailed participant information and seek consent.

For peer review only - http://bmjopen.bmj.com/site/about/guidelines.xhtml

Community pathway

Participants that have received either a new diagnosis, or a diagnosis within the last 12 months will also be approached through community groups, using a recruitment poster, invitation letter, reply slip, participant information leaflet, and freepost envelope. These participants will be contacted by a member of the research team by telephone at which time they will discuss the study and answer questions. The families will also be asked to consent to providing the original NHS diagnosis letter, which will be used by the research staff to determine eligibility to participate in the study, and legal guardianship at the first home visit.

Inclusion criteria

- Family includes child with ASD, aged 3-16 years
- Diagnosis of autism spectrum disorder, severity level 1 or 2
- Diagnosed within 12 months of consenting to the study
- If other diagnoses are present, ASD must be primary diagnosis
- Family are willing to comply with study requirements

Exclusion criteria

- Children with ASD severity level 3
- Children with ASD and intellectual impairment*
- Serious concomitant illness in child or family, or other circumstances such that they are unable to comply with study requirements
- Families who may be a risk to safety of research staff (This will be assessed by the clinical and research staff on the basis of clinical records, diagnosis letter and contact prior to the first home visit).
- Insufficient English language or capacity for parent/child to consent/assent to the study.

* Intellectual impairment will be identified by the clinical staff on the basis of pathway assessments described above including the WISC-V and the BAS3. Impairment will be

For peer review only - http://bmjopen.bmj.com/site/about/guidelines.xhtml

deemed present on the basis of any of the following criteria:

- The child has a comorbid diagnosis of intellectual disability
- Diagnosis specifies "with accompanying intellectual impairment"
- The child has been identified as requiring very substantial support (severity level 3) according to DSM-5 criteria for ASD
- The child is being educated in a special school for children with intellectual disabilities
- The child has an IQ of 70 or below

Study design

This is a randomised, controlled, multi-centred feasibility study including children with autism and their families (the Study Schema appears in Figure 1.) A total of 36 families will be recruited in four cohorts and each cohort will be randomised in a 2:1 ratio to receive support as usually employed (SUE) plus a programme of Systemic Autism-related Family Enabling (SAFE) therapy, or SUE alone, for a period of 16 weeks. Advantages of 2:1 allocation include:

- Increased appeal for patients deciding whether to consent to randomisation.
- Increased ability to test training of therapists, and ability to deliver high-fidelity treatment.
- Minimal reduction in statistical power for between-groups comparisons in a full-scale evaluation.
- Increased ability to recruit required number of families within an area before randomising; which will be closer to the figure needed if and when the intervention is implemented.

Outcome assessors will be blinded to allocation. All participants will complete outcome measures at baseline and again at 32 weeks post-allocation via a face-to-face visit, hence

For peer review only - http://bmjopen.bmj.com/site/about/guidelines.xhtml

each family will participate in the SAFE study for approximately eight months. An embedded qualitative study will collect information about the feasibility and acceptability of the intervention and the study itself. Qualitative data will be collected at a Family Feedback Day after the 32 week post-allocation visits have been completed. The end date for the trial will be the date on which the last family completes the Family Feedback Day.

Outcome measures

Feasibility outcome measures:

- Ability to identify, recruit and randomise eligible families.
- Acceptability of proposed outcome measures and follow-up schedule to participants, and whether targets for loss to follow-up are achievable.
- Adherence of therapists and families to the intervention.
- Ability to gather quantitative data on outcomes
- Appropriateness of resource use questionnaires and preference-based instruments for this population
- Effectiveness and scalability of training arrangements

Clinical outcome measures:

- Scores on the proposed primary outcome measure, the Systemic CORE 15 (SCORE) [45]. This is a 15 item paper-based survey, which has been shown to have good internal reliability (Cronbach's $\alpha = .89$) [46] and to be a valid index of family functioning, taking approximately 20 minutes to complete. The SCORE is the primary measure of family functioning employed in CYP (Children and Young People's) Improving Access to Psychological Therapies national programme, and is the gold standard for assessing the impact of family therapy on quality of life in the UK [47]. Every able family member will be asked to complete the SCORE, and the same

For peer review only - http://bmjopen.bmj.com/site/about/guidelines.xhtml

family members should complete the SCORE at baseline and 32 weeks. The SCORE-15 is freely available online [48].

- Scores on the proposed secondary outcome measures, which index changes in child behaviour, child-parent attachment, anxiety, and depression.
  - Patient Health Questionnaire – Somatic Anxiety Depressive Symptoms (PHQ-SADS). This comprises the PHQ-9 (estimated internal reliability Cronbach's α = .86-.89) [49] measuring depression and the GAD-7 (estimated internal reliability α =.92 [49] measuring anxiety [50].
  - Adapted mutuality sub-scale of the Coding of Attachment-Related Parenting for use with children with Autism - CARP-A [51]. The CARP-A is a validated observational measure of a child with autism's attachment behaviour towards their carer The CARP-A Mutuality subscale is reported as having inter-rater reliability of .74 [52].
  - The Child Behaviour Checklist (CBCL) [53]. This is a 30-item paper-based survey, which detects emotional and behavioural problems. Reasonable internal reliability is reported for each of three scales, given that some scales only have 4 items: 1. Competence scales (Cronbach's α = .63-.79) 2. Problems scales (Cronbach's α = .78-.97) and 3. DSM orientated scales (Cronbach's α = .72-.91) [54].
  - The Reflective Functioning Questionnaire (RFQ) [53] measures ability to understand own and others' mental states (Test-retest reliability coefficients are reported as 0.84) [55].
  - Caregiving Helplessness Questionnaire [56] (CGHQ). This is a 26-item questionnaire designed to assess aspects of disorganised caregiving. The CGHQ includes three subscales with reasonable internal reliability given the number of items: 1. Mother Helpless (α = .86) includes 7 items, 2. Mother-Child Frightened (α = .66) includes 6 items, 3. Child Caregiving (α = .64) includes 6 items [57].

For peer review only - http://bmjopen.bmj.com/site/about/guidelines.xhtml

- Scores on generic health economic outcome measures will also provide data on the feasibility of cost effectiveness analysis:
    - EuroQol 5 dimensions (EQ-5D-5L). This is a standardised generic instrument for measuring health outcome.
    - Child Health Utility 9 Dimensions (CHU-9D). The CHU-9D is a paediatric generic preference based measure of health related quality of life.
    - Resource Use Questionnaire (RUQ). A paper-based questionnaire completed by parent about his/her child's use of health care and social resources. The RUQ is designed to identify the NHS and Social Care resource use for the economic evaluation. It includes items to establish number and type of health resources being used, such as number of GP visits or number of days in hospital. Resource Use Questionnaire completion will be matched with medical records for a subgroup of families, which will help to develop strategies to minimise missing data in the future definitive trial.

- Qualitative outcomes:
    - Acceptability of SAFE and the trial process for participants and therapists
    - Reasons for declining and withdrawing from the study

The qualitative component will employ focus groups and interviews to investigate four key aspects of the study experience: families' experiences of the study (including intervention and potential harm of the intervention), therapists' experiences of the intervention, reasons for eligible families declining, and reasons for families withdrawing from the study. After the 32-week assessments have been completed, Families will be given details of the qualitative focus groups and invited to attend a family feedback day if they wish to do so. The family feedback day will involve several separate focus group sessions organised to take place over the period of a morning or an afternoon at a local venue for each centre including focus groups aimed specifically at parents and at children. The families will be told at the start of

For peer review only - http://bmjopen.bmj.com/site/about/guidelines.xhtml

the day that they are not obliged to respond to any question or prompt if they do not wish to and that the format of the day will be open discussion with other families in response to questions presented on a screen. They will then be invited to respond to a presented topic guide exploring the four key areas stated above through discussion with each other.

Place Figure 1. Here

The Intervention

SAFE is a manualised intensive programme of systemic family therapy designed to treat maladaptive autistic symptoms and mental health related difficulties encountered by families of children with autism. SAFE provides an array of therapeutic activities based on Attachment Theory, established systemic practice and the known visual processing preferences of people with autism [For example, 58,59,60]. SAFE is best seen as a toolkit with a variety of activities which can be applied to family therapy flexibly. For example, a very young child will engage with activities in a different way to teenagers. Activities include visual tasks, drawing, modelling, role-play and tracking circular patterns. Sessions are led by family need and the therapists and family work collaboratively, often in a playful way, utilising family resources, therapist expertise and the tools that SAFE provides. SAFE draws heavily from well-documented active and playful approaches in Family Therapy practice and literature [61].

Each therapy session will include two therapists with a minimum of intermediate family therapy level of qualification and four days training in SAFE principles. Prior to the therapy sessions parents allocated to SAFE will complete an adapted version of the Parent Development Interview [59], which will provide therapists with background information on family experience. The reflective functioning questions of this interview will also be revisited as an opportunity to discuss change at the 24-week follow up. Between weeks 1 and 16,

For peer review only - http://bmjopen.bmj.com/site/about/guidelines.xhtml

families allocated to the SAFE intervention will attend five 3-hour SAFE therapy sessions. Sessions 1 and 5 are multi-family sessions and will take place in a community setting. Sessions 2, 3 and 4 are for individual families and will take place in a community venue or the family home. The therapists will facilitate sessions which will be video recorded, as is usual practice for therapy sessions. The videotapes will be used by the therapists in supervision sessions and preparation for subsequent sessions.

Following completion of the therapy programme, families will attend a group follow-up session at 24 weeks post-allocation. Families will discuss any changes they have encountered focusing on their ability to be reflective about challenges faced and solutions tried. Trained support workers from local voluntary groups will attend this follow-up session and will be invited to give the families information about continued support for families of children with autism through existing networks.

Each session will include the following assessments for families to complete:

1. Client Satisfaction Questionnaire (CSQ-8)
2. The Helpful Aspects of Therapy Questionnaire (HAT)
3. A Between Session Activity (BSA) homework activity Families will be encouraged to complete a pro-forma with key elements of the intervention as prompts for families to track strengths and difficulties in response to SAFE ideas. Completion of the BSA will be recorded.

At the end of each session the therapists will also complete a training checklist and questionnaire to monitor protocol adherence.

Support as usually employed

Families will typically be offered a post-diagnosis follow-up appointment with the diagnosing

For peer review only - http://bmjopen.bmj.com/site/about/guidelines.xhtml

paediatrician. Parents of children whose symptoms are not severe may be directed to local authority parenting classes. Classes such as ‘Timeout for Autism’ (Cornwall and the Isles of Scilly) and ‘Understanding Autism’ (Plymouth) focus on the features of Autism Spectrum Disorder, instructional parenting techniques and issues associated with education. Psycho-education may also be offered, with families being directed to relevant resources for e.g. The National Autism Society, Gateway ASD, and the NHS Child and Adolescent Mental Health Services (CAMHS). For families where a member is experiencing depression or anxiety, treatment varies and is not linked to autism-related care. Initial referral is often through the GP. Patients may receive Cognitive Behavioural Therapy as part of the Improved Access for Psychological Therapies service. They may also receive medication and in extreme cases a period of in-patient hospital treatment.

(Place Figure 1 here)

Proposed sample size

In this feasibility trial no formal statistical testing of between group differences is planned. Sample size has been selected heuristically with the goal of i) demonstrating that participants can be recruited at a rate sufficient to run a full scale evaluation of SAFE at a later date; ii) demonstrating that it is possible to train therapists and deliver SAFE to patients within the study treatment settings, and iii) demonstrate that the data collection procedures are effective, and that the data collection is acceptable to the 36 families, and not overly burdensome.

Data analysis

Completed paper case report forms will be checked and signed by research staff before being sent to the Peninsula Clinical Trials Unit (CTU). Original case report form pages will be posted to the CTU at agreed time points for double-data entry on to a password-protected database, with copies retained at the study site. Forms will be tracked using a web-based trial management system. Data will be analysed and presented as is

For peer review only - http://bmjopen.bmj.com/site/about/guidelines.xhtml

appropriate for a feasibility study, in particular concentrating on descriptive analyses and undertaking no formal comparisons between groups. Reporting will follow the principles of the CONSORT Statement using the checklist and flowchart as recommended in the CONSORT extension for Randomized Pilot and Feasibility [62]. The flowchart will provide detail about the number of families approached, number eligible, number consenting, number randomised, number receiving allocated intervention and number assessed for outcome data at each time point. As appropriate, details will be given for individual members of the family, for example, how many family members there are and how many completed each questionnaire. Wherever possible, detailed reasons will be given for exclusions, loss to follow-up, non-completion of outcome measures etc.

Numbers will also be provided by centre and group, to inform the logistics of recruiting nine families prior to randomisation and following them up after randomisation. For those randomised to the SAFE intervention, adherence will be reported according to the number of group sessions attended and participation of individual family members at each of the therapy sessions. Completeness of data will be reported for each outcome measure at each relevant time point. Again this will be reported for individual family members as appropriate.

For each outcome measure, the relevant scores will be calculated and presented descriptively by trial arm. Where available, published guidelines will be used to process, score and summarise the measures including, for example, the use of imputation in the event of missing items on a questionnaire. Summary measures will be calculated as appropriate, for example, means and standard deviations, medians and ranges, numbers and percentages in categories. These measures will be presented both for baseline and for the final follow-up. The only analysis contrasting the two groups will be an interval estimate in the form of a 95% confidence interval for the primary outcome, so that the plausibility for the effect size used in the sample size calculation for the full trial can be assessed. For this purpose the baseline values will be used in an Analysis of Covariance, with acknowledgment that no effects are included for group or therapist.

For peer review only - http://bmjopen.bmj.com/site/about/guidelines.xhtml

Focus group interviews will be audio recorded and transcribed verbatim. Consequent qualitative data will be managed using proprietary computer assisted qualitative data analysis software, for example, Nvivo 10, and analysed thematically [63,64]. Rigour of analysis will include ‘respondent validation’, whereby participants are provided with a summary of their transcript and analysis so that they can assess whether the interpretations being made about the data, accurately represent them. In addition, a second qualitative researcher will conduct an independent analysis of a subset of half of the focus group transcripts. Researchers will then meet to discuss and agree the findings, which will then be presented to the Family Consultation Group for discussion.

Patient and Public Involvement

Families of children with autism initiated the development of this project by communicating their complex needs and dissatisfaction with current service provision through the Plymouth Autism Network. This Network was set up by the Chief Investigator in 2011 to bring clinicians, carers, academics and individuals with autism together to share ideas, research findings and experiences. We further explored the challenges facing families of children with autism by conducting in-depth interviews and surveying over 90 families regarding their needs and the treatment they received post-diagnosis. Less than 9% of families agreed that current treatment helped with the problems they face. Our survey revealed a strong need for interventions, which support the whole family.

This pilot data led to the development of a research team within the Welcome Research Hub at Plymouth University, which included a Family Consultation Group. Our Family Consultation Group worked with us to develop and refine the SAFE intervention prior to the current project. These families have also contributed to the creation of a recruitment and treatment plan, which will be manageable for families. They have offered advice about how it is best to communicate with families at the start of the study and as it progresses. In addition, the Family Consultation Group representative is a co-applicant on this

For peer review only - http://bmjopen.bmj.com/site/about/guidelines.xhtml

study.

Our Family Consultation Group will continue to be essential members of the team and work as an advisory group throughout the feasibility study and beyond. Formal structures are in place to ensure ongoing collaboration with the Family Consultation Group. Specifically, the representative for the Consultation Group is paid as a research assistant on the trial and is a co-applicant. She attends and actively contributes to monthly trial management group meetings, all training sessions and fortnightly research team meetings. The representative reports key issues and requests to and from the wider group. Where necessary, additional meetings are held between the family Consultation Group as a whole and other research staff. In these instances travel and subsistence costs are available in line with National Health Service England guidelines on working with our patient and public voice partners [65].

We see our Family Consultation Group as experts in their own lives and the lives of families with similar challenges. For this reason we feel our role is to work with them in a supportive manner as collaborators. Their contribution is valuable in the same way as other experts on the team and we aim to facilitate one another. As stated above, the Family Consultation Group have been active in contributing to the research plan. Their input is of particular value in developing recruitment procedures, designing participant information packs and providing information about potential barriers to retention. We have also worked with them to prepare and deliver a training programme for research staff and therapists. With their help we have trained recruited staff work in a sensitive and informed manner with participants. We also value their input in interpreting and reporting data; in particular commenting on possible ways to overcome challenges for the main RCT.

Our families can help by identifying local networks and sharing their experience with new groups. Our Family Consultation Group are proactive campaigners for change and have extensive knowledge of existing bodies such as the National Autistic Society. They can also

For peer review only - http://bmjopen.bmj.com/site/about/guidelines.xhtml

provide a family-centred perspective on research outcomes. They are, therefore, well-placed to collaborate with us in planning next steps and disseminating findings at local and national levels.

ETHICS AND DISSEMINATION

Risks and safety

Families of children with autism are a potentially vulnerable group. The risks associated with participating in this study are however, considered minimal, with no adverse events anticipated in any participant. For those in the intervention group, there is a slight chance that the SAFE family therapy sessions could lead to an initial increase in family disagreements as family members learn how to change the way they solve problems and talk with one another. However, the purpose of the intervention is ultimately to equip families with skills to handle these difficulties by learning how to change the way they solve problems and improve their communication, and the SAFE family therapists will be available to provide support and will be trained to handle any emerging problems. Should any issues arise the SAFE family therapists will have access to two consultant clinical psychologists to provide further support and advice.

During the trial the children with autism will remain in the care of the Child Development Centre or the Autism Spectrum Disorder Assessment Team and will have access to usual care should any unforeseen circumstances arise. Other members of the family will also continue to be able to seek care and advice from the GP or any other specialist services they are concurrently involved with.

Monitoring adverse events

The research team have mechanisms in place to report serious adverse events (SAE) related to mental health. Serious Adverse Events related to mental health may be

For peer review only - http://bmjopen.bmj.com/site/about/guidelines.xhtml

volunteered by the participant or discovered by the therapists, research assistants or other member of the research team during the SAFE family therapy sessions, or as a result of direct reporting (e.g. by telephone) by a family member, independent clinician or other informant. Serious adverse events will be recorded from the time of consent until the date the participant completes the follow-up or withdraws from the study.

If the CI considers that the SAE is not, or is unlikely to be, associated with the trial, the CTU will obtain a second assessment of causality from an independent assessor. Any SAE which in the opinion of either adjudicator is possibly related to the trial will be reported to the Research Ethics Committee within 15 days of the local research team having become aware of the event. All SAEs will be followed until either stabilised if chronic conditions or resolved.

Dissemination

If the feasibility study meets progression criteria an important part of our dissemination plan is to raise awareness of the need for a larger multi-centre trial. We will, therefore, offer targeted summaries of our findings and presentations to policy makers. The findings will also be broadly disseminated, but in a manner appropriate to a feasibility study. We plan national conference presentations and published papers to inform clinicians, academics and therapists about the possible benefits of SAFE and generate interest in the future trial. We will make use of our existing connections including the Association for Family Therapy, the National Autistic Society and the Institute of Family Therapy to reach relevant audiences. Our qualitative findings will also be published with detailed accounts of the families' reactions to SAFE and their views on its effectiveness. We will also provide forums for participating families to share their own experiences of the intervention with wider audiences through existing networks, groups and events across the UK.

Our Family Consultation Group will be integral to our dissemination plan. Their involvement will include presenting their experiences as delegates at national and international

For peer review only - http://bmjopen.bmj.com/site/about/guidelines.xhtml

conferences, being active co-authors on published papers, leading the organising committee for a local event sharing findings with families, key local stakeholders, clinicians and other interested partners; and liaising with other bodies to raise awareness of the study findings including Autistica, The National Autistic Society and the Brandon Trust.

Informing potential participants of possible benefits and known risks

The participant information sheets and leaflets will provide potential participants with information about the possible benefits and risks of taking part in the trial. For example, the participants will be informed that a potential risk of receiving the SAFE therapy is that the sessions may evoke difficult emotions and feelings this could lead to family disagreement as they move towards change. The families will also be informed that benefits of the trial include the possibility of improved coping skills when faced with challenges and contribution to finding out if SAFE can progress to a national trial. Participants will be given the opportunity to discuss risks and benefits with a member of the research team prior to consenting to participate.

Obtaining Informed consent from participants

All participants will receive a leaflet and information sheet prior to consent. There are two versions of the information sheet, one for adults and one for children. In the leaflet parents are encouraged to explain the trial to their younger children and some guidance for doing this is provided. The information sheet states that the participants have the right to withdraw at any point during the trial and that data collected from them will be confidential. All participants will have a home-visit prior to consent from a member of the research team and will be able to ask questions or go through the information verbally. Participants will have the process of the study explained to them including the estimated time they will have to wait prior to randomisation and starting the intervention if allocated to that arm.

For peer review only - http://bmjopen.bmj.com/site/about/guidelines.xhtml

Data protection/confidentiality

Participants will be given a unique identification number. The data will be pseudo-anonymised in the sense that there will be an identification number on the documentation but otherwise no means of identifying the individual to which the data relates. The research team will ensure that participants' pseudo-anonymity is maintained on all documents. Data will be collected and stored in accordance with the current legal and regulatory documentation. Electronic study records will be stored in a SQL server database, stored on a restricted access, secure server maintained by Plymouth University. Data will be entered into the database via a bespoke web-based data entry system encrypted using SSL. Access to electronic data will be permission based, and at the discretion of the clinical trials unit data management team.

Anonymised paper-based study data will be stored in locked filing cabinets. Copies of study data retained at the lead study site will be securely stored for the duration of the study prior to archiving. Video data will be transported via encrypted memory sticks and will be transferred to a password-protected computer. The clinical trials unit data team will have access to study data, including identifiable data. Other members of the study team and the trials unit will have restricted access to pseudo-anonymised study data. Access will be granted to the Sponsor and host institution on request, to permit study-related monitoring, audits and inspections.

Research governance and the conduct of the trial

The trial will be conducted to protect the human rights and dignity of the participant as reflected in the Helsinki Declaration. An important factor in protecting the participants is ongoing consultation with the SAFE Family Consultation Group. A representative of this group is a member of the research team and is involved in decision-making processes. The research team including the family consultation group are proactive in minimising

For peer review only - http://bmjopen.bmj.com/site/about/guidelines.xhtml

discomfort and risk for participants, respecting their wishes over science and society, respecting the right to withdraw and the need for families to have access to all relevant information.

The Chief Investigator will be responsible for the overall conduct of the study, keeping it to schedule and within budget. Working closely with the CTU she will be the focal contact for enquiries from both sites. The CTU will manage the study, liaise with sites, monitor recruitment, work with the Sponsor and report to Trial Management Group (TMG) meetings. The TMG will meet regularly throughout the feasibility study. A Trial Steering Committee (TSC) will have an overarching monitoring responsibility. The TSC is expected to meet three times during the study, but will be additionally convened at the chairman or Chief Investigator's request.

Dissemination plans

If the feasibility study demonstrates successful recruitment, data collection and an ability to deliver the intervention, an important part the dissemination plan is to raise awareness of the need for a larger multi-centre trial. Targeted summaries of the findings and presentations will be disseminated to policy makers. The findings will also be broadly disseminated, but in a manner appropriate to a feasibility study. National conference presentations and published papers will be prepared to inform clinicians, academics and therapists about our feasibility results and generate interest in the future trial. Existing connections including the Association for Family Therapy, the National Autistic Society and the Institute of Family Therapy will be utilised to reach relevant audiences. The qualitative findings will also be published with detailed accounts of the families' reactions to SAFE and their views on its usefulness. A summary of study results in plain English will be available on the Peninsula Clinical Trials website.

For peer review only - http://bmjopen.bmj.com/site/about/guidelines.xhtml

Clinical trials authorisation and ethical approval

Clinical trials authorisation is not required. The study has appropriate Research Ethics Committee (REC) approval from the South West-Exeter Research Ethics Committee (Ref: 17/SW/0192) and approval from the Health Research Authority (HRA). The trial will be conducted in accordance with the protocol, the principles of the Declaration of Helsinki and ICH GCP. Any amendments of the protocol will be submitted to the Sponsor, HRA and REC for approval.

Trial sponsorship

The trial is sponsored by University Hospitals Plymouth NHS Trust.

Trial Steering Committee

The Trial Steering Committee will include an independent chair and at two other independent members, along with the lead investigator and the other study collaborators including a parent representative. They will meet once a year.

REFERENCES

1. National Autistic Society, 'Autism Facts and History', accessed 22 March 2018

2. American Psychiatric Association. Diagnostic and Statistical Manual of Mental Disorders, 5th edn. American Psychiatric Association, Washington, DC 2013.

3. Sasson NJ, Lam KSL, Parlier M, et al. Autism and the broad autism phenotype: familial patterns and intergenerational transmission. *J Neurodev Disord* 2013; 5:11-18.

4. Dykens EM, Fisher MH, Taylor JL, et al. Reducing Distress in Mothers of Children with Autism and Other Disabilities: A Randomized Trial. *Pediatrics* 2014; 2013-3164.

For peer review only - http://bmjopen.bmj.com/site/about/guidelines.xhtml

5. Hallett V, Ronald A, Colvert, E, et al. Exploring anxiety symptoms in a large-scale twin study of children with autism spectrum disorders, their co-twins and controls. *Journal of Child Psychology and Psychiatry* 2013;54:1176-1185.

6. Brereton AV, Tonge BJ, Einfeld SL. Psychopathology in children and adolescents with autism compared to young people with intellectual disability. *J Autism Dev Disord* 2006;36: 863-870.

7. Kim JA, Szatmari P, Bryson SE, et al. The prevalence of anxiety and mood problems among children with autism and Asperger syndrome. *Autism* 2000; 4:117-132.

8. Howlin P. Interventions for people with autism: recent advances*. Adv Psychiatr Treat* 1997;3: 94-102.

9. Mazefsky CA, Folstein SE, Lainhart JE. Overrepresentation of Mood and Anxiety Disorders in Adults with Autism and Their First Degree Relatives: What Does it Mean? *Autism Res* 2008;1:193-197.

10. Daniels JL, Forssen U, Hultman M, et al. Parental Psychiatric Disorders Associated With Autism Spectrum Disorders in the Offspring. *Pediatrics* 2008;121:1357-1162.

11. Siklos S, Kerns KA. Assessing Need for Social Support in Parents of Children with Autism and Down Syndrome. *J Autism Dev Disord* 2008;36:921-933.

12. Markoulakis R, Fletcher P, Bryden P. Seeing the glass half full: benefits to the lived experiences of female primary caregivers of children with autism. *Clin Nurse Spec.* 2012;26(1):48–56.

13. Potter CA. ‘I accept my son for who he is – he has incredible character and personality’: fathers’ positive experiences of parenting children with autism. *Disabil Soc,* 2016;31(7):948-965.

For peer review only - http://bmjopen.bmj.com/site/about/guidelines.xhtml

14. Myers BJ, Mackintosh VH, Goin-Kochel RP. “My greatest joy and my greatest heart ache:” Parents’ own words on how having a child in the autism spectrum has affected their lives and their families’ lives. *Res Autism Spectr Disord.* 2009;3(3):670-684.

15. Zhang W, Yan TT, Barriball KL, While AE, Liu XH. Post-traumatic growth in mothers of children with autism: a phenomenological study. *Autism.* 2015;19(1):29-37.

16. Altiere MJ, von Kluge S. Searching for acceptance: challenges encountered while raising a child with autism. *J Intellect Dev Disabil.* 2009;34(2):142-152.

17. McCrone P, Dhanasiri S, Patel A, et al. The cost of mental health care in England to 2026. Executive Summary, London: The King’s Fund. 2008

18. Buescher AVS, Cidav Z, Knapp M, et al. Costs of Autism Spectrum Disorders in the United Kingdom and the United States. *JAMA Pediatr* 2014;e1-e8.

19. Osborne LA, McHugh L, Saunders J, et al. Parenting Stress Reduces the Effectiveness of Early Teaching Interventions for Autistic Spectrum Disorders. *J Autism Dev Disord* 2008;38:1092-1103.

20. Pisula E. Parenting Stress in Mothers and Fathers of Children with Autism Spectrum Disorders, A Comprehensive Book on Autism Spectrum Disorders, Dr. Mohammad-Reza Mohammadi (Ed.) 2011. Available from: http://www.intechopen.com/books/a-comprehensive- bookon- autism-spectrum-disorders/parenting-stress-in-mothers-and-fathers-of-children-with-autismspectrum-disorders

21. Nikmat AW, Ahmad M, Oon NL, et al. Stress and psychological wellbeing among parents of children with autism spectrum disorder. *ASEAN Journal of Psychiatry* 2008;9:65-72.

For peer review only - http://bmjopen.bmj.com/site/about/guidelines.xhtml

22. Bolton PF, Pickles A, Murphy M, et al. Autism, affective and other psychiatric disorders: patterns of familial aggregation. *Psychological Medicine* 1998;28: 385-395.

23. Demir T, Dogangun B, Karacetin G, et al. Psychiatric Disorders and Symptoms in Mothers of Children with Autistic Disorder. *Neurol Psychiatry Brain Res* 2008;15:1-5.

24. Piven J, Palmer P. Psychiatric disorder and the broad autism phenotype: evidence from a family study of multiple-incidence autism families. *Am J Psychiatry* 1999;156:557-563.

25. Roberts AL, Koenen KC, Lyall K, Ascherio A, Weisskopf MG. Women's posttraumatic stress symptoms and autism spectrum disorder in their children. *Res Autism Spectr Disord* 2014;8(6):608-616.

26. Roberts AL, Lyall K, Rich-Edwards JW, Ascherio A, Weisskopf MG. Maternal exposure to intimate partner abuse before birth is associated with autism spectrum disorder in offspring. *Autism* 2016;20(1):26-36.

27. Konstantareas MK. Autistic, learning disabled and delayed children's impact on their parents. *Can J Behav Sci* 1991;23:358-375.

28. Murphy M, Bolton PF, Pickles A, et al. Personality traits of the relatives of autistic probands. *Psychol Med* 2000;30:1411-1424.

29. Blackledge JT, Hayes SC. Using Acceptance and Commitment Training in the Support of Parents of Children Diagnosed with Autism. *Child & Family Behavior Therapy,* 2006:28:1-18.

30. NICE guidelines [CG170] https://www.nice.org.uk/guidance/cg170/chapter/patient-centred-care

31. NICE guidelines [CG128] https://www.nice.org.uk/guidance/cg128

For peer review only

For peer review only - http://bmjopen.bmj.com/site/about/guidelines.xhtml

32. Munroe E. The Munroe Review of Child protection: Final report, Dept. of Education, HM Stationary Office, UK 2011.

33. Goodman S, Trowler I. Social work re-claimed: Innovative frameworks for child and social work Practice. London: Jessica Kingsley 2012.

34. NHS England. Future in Mind: promoting, protecting and improving our children and young people's mental health and wellbeing. Dept of Health Publication Gateway Ref. No 02939, 2015.

35. IAPT | CYP IAPT Children and Young People's Project. (n.d.). Retrieved January 5, 2015, from http://www.iapt.nhs.uk/cyp-iapt/

36. Bromley J, Hare DJ, Davison K. et al. Mothers supporting children with autistic spectrum disorders: Social support, mental health status and satisfaction with services. *Autism* 2004;8:409-423.

37. Stratton P. Report On The Evidence Base Of Systemic Family Therapy. *Association for Family Therapy* 2005.

38. Dallos R, Draper R. (4th Edn) An Introduction to Family Therapy. Maidenhead: Open Univ. Press/McGraw Hill 2015.

39. Monteiro M. Family Therapy and the Autism Spectrum: Autism Conversations in Narrative Practice. London: Routledge 2016.

40. Estrada AU, Pinsof WF. The effectiveness of family therapies for selected behavioural disorders of childhood. *J Marital Fam Ther* 1995;21: 403-424.

41. Pellicano E, Dinsmore A, Charman T. What should autism research focus upon? Community views and priorities from the United Kingdom. *Autism, Special Issue Article* 2014:1-15.

For peer review only

For peer review only - http://bmjopen.bmj.com/site/about/guidelines.xhtml

42. Wallace S, Parr J, Hardy, A. One in a hundred: Putting families at the heart of autism research. London: Autistica 2013.

43. Wechsler D. WISC-V: Technical and Interpretive Manual. NCS Pearson, Incorporated; 2014.

44. Eliot CD, Smith P. British Ability Scales , BAS3. London, United Kingdom: GL Assessment. 2011.

45. Stratton P, Bland J, Janes E, et al. Developing an indicator of family function and a practicable outcome measure for systemic family and couple therapy: the SCORE. *Journal of Family Therapy* 2010;32:232-258.

46. Stratton P, Lask J, Bland J, Nowotny E, Evans C, Singh R, Janes E, Peppiatt A. Detecting therapeutic improvement early in therapy: validation of the SCORE-15 index of family functioning and change. *J Fam Ther* 2014;36:3-19.

47. SHIFT Self-Harm Intervention, Family Therapy: a randomised controlled trial of family therapy vs. treatment as usual for young people seen after second or subsequent episodes of self harm. Protocol Grant Number: 470772 HTA Reference Number: 07/33/01, 2015.

48. SCORE-15 [Internet] Association for Family Therapy www.aft.org.uk. [cited 2018 Sept 17] Available from: http://www.aft.org.uk/SpringboardWebApp/userfiles/aft/file/SCORE%20info/SCORE-15%20102013.pdf

49. Kroenke K, Spitzer RL, Williams JBW, Lowe B. The Patient Health Questionnaire Somatic, Anxiety, and Depressive Symptom Scales: a systematic review. *Gen Hosp Psychiatry* 2010;32:345-359.

For peer review only - http://bmjopen.bmj.com/site/about/guidelines.xhtml

50. Spitzer R L, Kroenke K, Williams JBW. The Patient Health Questionnaire Primary Care Study Group. Validation and utility of a self-report version of PRIME-MD: the PHQ Primary Care Study. *JAMA,* 282, 1999:1737-1744.

51. Blazey L. Coding of Attachment-Related Parenting for use with children with Autism (CARP-A) [dissertation]. Manchester: Manchester University, 2007.

52. Holt CE. Child-caregiver Play and Attachment in Pre-school Autism: A Longitudinal Investigation [thesis]. Manchester: University of Manchester, 2011.

53. Moss E, Bureau J, Cyr C, et al. Correlates of attachment at age 3 Construct validity of the Preschool Attachment Classification System. *Dev. Psychol* 2004;40:323-334.

54. Achenbach TM, Rescorla LA. Manual for the ASEBA School-Age Forms and Profiles: Child Behaviour Checklist for Ages 6-18. Burlington, VT: University of Vermont, Research Center for Children, Youth, & Families. 2001.

55. Fonagy P, Luyten P, Moulton-Perkins A, et al. Development and validation of a self-report measure of mentalizing: The Reflective Functioning Questionnaire. PLOS ONE. 2016;11(7):e0158678.

56. Solomon J, George C. (Eds.) Disorganised Attachment and Caregiving. Guilford: New York; 2011.

57. Huth-Bocks AC, Guyon-Harris K, Calvert M, Scott S, Ahlfs-Dunn S. The Caregiving Helplessness Questionnaire: Evidence for validity and utility with mothers of infants. *Infant Ment Health J.* 2016;37:208-221.

57. Ogletree BT, Morrow-Odom KL, Wrestling D. Understanding the brain–behaviour relationship in persons with ASD: Implications for PECS as a treatment choice. *Dev Neurorehabil* 2015;18(2): 88-96.

For peer review only - http://bmjopen.bmj.com/site/about/guidelines.xhtml

58. Buron KD, Wolfberg P. Learners on the autism spectrum: Preparing highly qualified educators (pp. 114–137). AAPC Publishing: Shawnee; 2008.

59. Grandin T, Scariano MM. (1986). Emergence: Labeled autistic. New York, NY: Warner Books 1986.

60. Slade A, Aber JL, Bresgi I, Berger B, Kaplan M. The parent development interview, revised. Unpublished protocol. The City University of New York 2004.

61. Dallos R, Draper R. An introduction to family therapy: Systemic theory and practice. McGraw-Hill Education (UK); 2015.

62. CONSORT extension for Randomized Pilot and Feasibility Trials (http://www.consort-statement.org/randomised-pilot-and-feasibilitytrials).

63. Braun V, Clarke V. Using thematic analysis in psychology. *Qual Res Psychol.* 2006;3:77-101.

64. Green J, Thorogood N. Qualitative Health Methods for health Research. London: Sage Publications; 2004.

65. NHS ENGLAND. Working with our Patient and Public Voice (PPV) Partners – Reimbursing expenses and paying involvement payments. 2017.

For peer review only

AUTHORS' CONTRIBUTIONS: RM, JV and HH were responsible for the overall development of the protocol. RM, HH, RD, CM, PE, AB, TV and the Family Consultation Group were involved in the conception and production of the study and the development of the initial protocol. PE and AB provided methodological expertise and advice on quantitative analysis PE provided statistical expertise. RD was the lead researcher, with the support of

For peer review only - http://bmjopen.bmj.com/site/about/guidelines.xhtml

JS, on design of the intervention and the qualitative component. TV, with the support of the Family Consultation Group, and CM advised on design and ethics, particularly from the participant perspective. All authors made substantial contributions to drafting, revision and approval of the document.

ACKNOWLEDGEMENTS: The Authors would like to thank the SAFE Family Consultation Group for their ongoing input and expertise. They would also like to thank James Cook, Mary Hosken, Dr Ben Whalley, Dr Antonieta Medina-Lara and Professor Julian Archer for their help in developing and conducting this study. The authors would also like to give special recognition to the dedication, hard-work and sensitivity of Helen Hancocks, the SAFE trial manager for the Peninsula Clinical Trials Unit.

FUNDING STATEMENT: This work was supported by the National Institute for Health Research (NIHR) grant number: PB-PG-0815-20058

COMPETING INTERESTS STATEMENT: Contributors are co-applicants or employed research staff on the SAFE project, which receives funds from both NIHR and Autistica. Professor Rudi Dallos holds joint Intellectual Property rights for the SAFE intervention with The University of Plymouth. No other issues were raised with regard to competing interests.

PROVENANCE AND PEER REVIEW: Not commissioned; externally peer reviewed.

DATA SHARING STATEMENT: Further details of the study protocol can be requested from the corresponding author.

Caption for Figure 1:

Figure 1. Study Schema

For peer review only - http://bmjopen.bmj.com/site/about/guidelines.xhtml

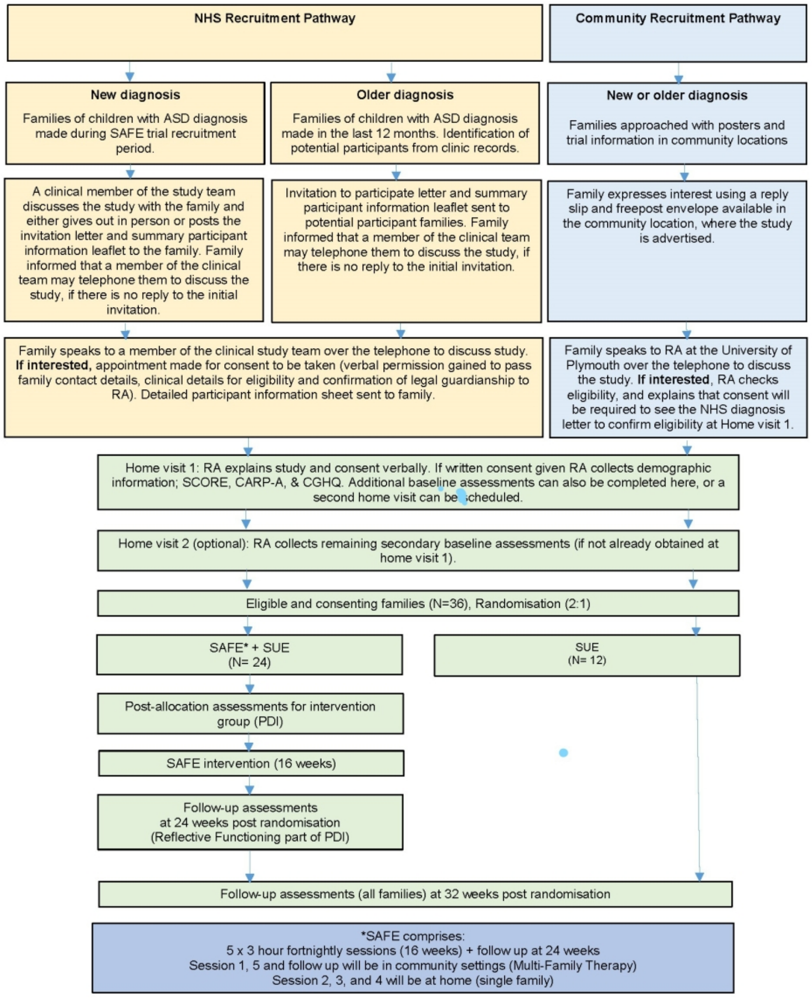
NHS Recruitment Pathway
Community Recruitment Pathway
New diagnosis
Families of children with ASD diagnosis made during SAFE trial recruitment period.
Older diagnosis
Families of children with ASD diagnosis made in the last 12 months. Identification of potential participants from clinic records.
New or older diagnosis
Families approached with posters and trial information in community locations
A clinical member of the study team discusses the study with the family and either gives out in person or posts the invitation letter and summary participant information leaflet to the family. Family informed that a member of the clinical team may telephone them to discuss the study, if there is no reply to the initial invitation.
Invitation to participate letter and summary participant information leaflet sent to potential participant families. Family informed that a member of the clinical team may telephone them to discuss the study, if there is no reply to the initial invitation.
Family expresses interest using a reply slip and freepost envelope available in the community location, where the study is advertised.
Family speaks to a member of the clinical study team over the telephone to discuss study. If interested, appointment made for consent to be taken (verbal permission gained to pass family contact details, clinical details for eligibility and confirmation of legal guardianship to RA). Detailed participant information sheet sent to family.
Family speaks to RA at the University of Plymouth over the telephone to discuss the study. If interested, RA checks eligibility, and explains that consent will be required to see the NHS diagnosis letter to confirm eligibility at Home visit 1.
Home visit 1: RA explains study and consent verbally. If written consent given RA collects demographic information; SCORE, CARP-A, & CGHQ. Additional baseline assessments can also be completed here, or a second home visit can be scheduled.
Home visit 2 (optional): RA collects remaining secondary baseline assessments (if not already obtained at home visit 1).
Eligible and consenting families (N=36), Randomisation (2:1)
SAFE* + SUE
(N= 24)
SUE
(N= 12)
Post-allocation assessments for intervention group (PDI)
SAFE intervention (16 weeks)
Follow-up assessments
at 24 weeks post randomisation
(Reflective Functioning part of PDI)
Follow-up assessments (all families) at 32 weeks post randomisation
*SAFE comprises:
5 x 3 hour fortnightly sessions (16 weeks) + follow up at 24 weeks
Session 1, 5 and follow up will be in community settings (Multi-Family Therapy)
Session 2, 3, and 4 will be at home (single family)

SPIRIT

STANDARD PROTOCOL ITEMS: RECOMMENDATIONS FOR INTERVENTIONAL TRIALS

SPIRIT 2013 Checklist: Recommended items to address in a clinical trial protocol and related documents*

Section/item	Item No	Description	Addressed on page number
**Administrative information**			
Title	1	Descriptive title identifying the study design, population, interventions, and, if applicable, trial acronym	Front page
Trial registration	2a	Trial identifier and registry name. If not yet registered, name of intended registry	Front page
	2b	All items from the World Health Organization Trial Registration Data Set	Front page, 6, 9
Protocol version	3	Date and version identifier	Front page
Funding	4	Sources and types of financial, material, and other support	34
Roles and responsibilities	5a	Names, affiliations, and roles of protocol contributors	6
	5b	Name and contact information for the trial sponsor	6
	5c	Role of study sponsor and funders, if any, in study design; collection, management, analysis, and interpretation of data; writing of the report; and the decision to submit the report for publication, including whether they will have ultimate authority over any of these activities	5
	5d	Composition, roles, and responsibilities of the coordinating centre, steering committee, endpoint adjudication committee, data management team, and other individuals or groups overseeing the trial, if applicable (see Item 21a for data monitoring committee)	30 - 32

For peer review only - http://bmjopen.bmj.com/site/about/guidelines.xhtml

**Introduction**			
Background and rationale	6a	Description of research question and justification for undertaking the trial, including summary of relevant studies (published and unpublished) examining benefits and harms for each intervention	10 -11
	6b	Explanation for choice of comparators	29 - 30
Objectives	7	Specific objectives or hypotheses	11 - 12
Trial design	8	Description of trial design including type of trial (eg, parallel group, crossover, factorial, single group), allocation ratio, and framework (eg, superiority, equivalence, noninferiority, exploratory)	12
**Methods: Participants, interventions, and outcomes**			
Study setting	9	Description of study settings (eg, community clinic, academic hospital) and list of countries where data will be collected. Reference to where list of study sites can be obtained	16
Eligibility criteria	10	Inclusion and exclusion criteria for participants. If applicable, eligibility criteria for study centres and individuals who will perform the interventions (eg, surgeons, psychotherapists)	16
Interventions	11a	Interventions for each group with sufficient detail to allow replication, including how and when they will be administered	21 - 22
	11b	Criteria for discontinuing or modifying allocated interventions for a given trial participant (eg, drug dose change in response to harms, participant request, or improving/worsening disease)	21
	11c	Strategies to improve adherence to intervention protocols, and any procedures for monitoring adherence (eg, drug tablet return, laboratory tests)	21, 30
	11d	Relevant concomitant care and interventions that are permitted or prohibited during the trial	22
Outcomes	12	Primary, secondary, and other outcomes, including the specific measurement variable (eg, systolic blood pressure), analysis metric (eg, change from baseline, final value, time to event), method of aggregation (eg, median, proportion), and time point for each outcome. Explanation of the clinical relevance of chosen efficacy and harm outcomes is strongly recommended	14 - 15
Participant timeline	13	Time schedule of enrolment, interventions (including any run-ins and washouts), assessments, and visits for participants. A schematic diagram is highly recommended (see Figure)	19

For peer review only - http://bmjopen.bmj.com/site/about/guidelines.xhtml

Sample size	14	Estimated number of participants needed to achieve study objectives and how it was determined, including clinical and statistical assumptions supporting any sample size calculations	29 - 30
Recruitment	15	Strategies for achieving adequate participant enrolment to reach target sample size	20
**Methods: Assignment of interventions (for controlled trials)**			
Allocation:			
Sequence generation	16a	Method of generating the allocation sequence (eg, computer-generated random numbers), and list of any factors for stratification. To reduce predictability of a random sequence, details of any planned restriction (eg, blocking) should be provided in a separate document that is unavailable to those who enrol participants or assign interventions	29 - 30
Allocation concealment mechanism	16b	Mechanism of implementing the allocation sequence (eg, central telephone; sequentially numbered, opaque, sealed envelopes), describing any steps to conceal the sequence until interventions are assigned	19 - 20
Implementation	16c	Who will generate the allocation sequence, who will enrol participants, and who will assign participants to interventions	20
Blinding (masking)	17a	Who will be blinded after assignment to interventions (eg, trial participants, care providers, outcome assessors, data analysts), and how	20 - 21
	17b	If blinded, circumstances under which unblinding is permissible, and procedure for revealing a participant's allocated intervention during the trial	20 - 21
**Methods: Data collection, management, and analysis**			
Data collection methods	18a	Plans for assessment and collection of outcome, baseline, and other trial data, including any related processes to promote data quality (eg, duplicate measurements, training of assessors) and a description of study instruments (eg, questionnaires, laboratory tests) along with their reliability and validity, if known. Reference to where data collection forms can be found, if not in the protocol	27 - 29
	18b	Plans to promote participant retention and complete follow-up, including list of any outcome data to be collected for participants who discontinue or deviate from intervention protocols	24 - 25

For peer review only - http://bmjopen.bmj.com/site/about/guidelines.xhtml

Data management	19	Plans for data entry, coding, security, and storage, including any related processes to promote data quality (eg, double data entry; range checks for data values). Reference to where details of data management procedures can be found, if not in the protocol	28
Statistical methods	20a	Statistical methods for analysing primary and secondary outcomes. Reference to where other details of the statistical analysis plan can be found, if not in the protocol	29 - 30
	20b	Methods for any additional analyses (eg, subgroup and adjusted analyses)	25 - 26
	20c	Definition of analysis population relating to protocol non-adherence (eg, as randomised analysis), and any statistical methods to handle missing data (eg, multiple imputation)	29 - 30
**Methods: Monitoring**			
Data monitoring	21a	Composition of data monitoring committee (DMC); summary of its role and reporting structure; statement of whether it is independent from the sponsor and competing interests; and reference to where further details about its charter can be found, if not in the protocol. Alternatively, an explanation of why a DMC is not needed	31 - TSC performing DMC role.
	21b	Description of any interim analyses and stopping guidelines, including who will have access to these interim results and make the final decision to terminate the trial	33
Harms	22	Plans for collecting, assessing, reporting, and managing solicited and spontaneously reported adverse events and other unintended effects of trial interventions or trial conduct	26 - 27
Auditing	23	Frequency and procedures for auditing trial conduct, if any, and whether the process will be independent from investigators and the sponsor	30 - 31
**Ethics and dissemination**			
Research ethics approval	24	Plans for seeking research ethics committee/institutional review board (REC/IRB) approval	32 - 33
Protocol amendments	25	Plans for communicating important protocol modifications (eg, changes to eligibility criteria, outcomes, analyses) to relevant parties (eg, investigators, REC/IRBs, trial participants, trial registries, journals, regulators)	32

For peer review only - http://bmjopen.bmj.com/site/about/guidelines.xhtml

Consent or assent	26a	Who will obtain informed consent or assent from potential trial participants or authorised surrogates, and how (see Item 32)	18
	26b	Additional consent provisions for collection and use of participant data and biological specimens in ancillary studies, if applicable	23
Confidentiality	27	How personal information about potential and enrolled participants will be collected, shared, and maintained in order to protect confidentiality before, during, and after the trial	28 - 29
Declaration of interests	28	Financial and other competing interests for principal investigators for the overall trial and each study site	________
Access to data	29	Statement of who will have access to the final trial dataset, and disclosure of contractual agreements that limit such access for investigators	29
Ancillary and post-trial care	30	Provisions, if any, for ancillary and post-trial care, and for compensation to those who suffer harm from trial participation	33
Dissemination policy	31a	Plans for investigators and sponsor to communicate trial results to participants, healthcare professionals, the public, and other relevant groups (eg, via publication, reporting in results databases, or other data sharing arrangements), including any publication restrictions	33 - 34
	31b	Authorship eligibility guidelines and any intended use of professional writers	________
	31c	Plans, if any, for granting public access to the full protocol, participant-level dataset, and statistical code	________
**Appendices**			
Informed consent materials	32	Model consent form and other related documentation given to participants and authorised surrogates	________
Biological specimens	33	Plans for collection, laboratory evaluation, and storage of biological specimens for genetic or molecular analysis in the current trial and for future use in ancillary studies, if applicable	N/A

*It is strongly recommended that this checklist be read in conjunction with the SPIRIT 2013 Explanation & Elaboration for important clarification on the items. Amendments to the protocol should be tracked and dated. The SPIRIT checklist is copyrighted by the SPIRIT Group under the Creative Commons "Attribution-NonCommercial-NoDerivs 3.0 Unported" license.

For peer review only - http://bmjopen.bmj.com/site/about/guidelines.xhtml
